# Ubiquitination of Rhomboid 5 Homolog 2 by Constitutive Photomorphogenic 1 Alleviates Hepatic Ischemia-reperfusion Injury by Regulating the Transforming Growth Factor-β Activating Kinase 1-C-Jun N-terminal Kinase/p38 Signaling Pathway

**DOI:** 10.1016/j.jcmgh.2025.101695

**Published:** 2025-12-05

**Authors:** Wendong Li, Tongtong Wu, Hao Li, Zhenyu Guan, Mingjie Ding, Wenzhi Guo

**Affiliations:** 1 Department of Hepatobiliary and Pancreatic Surgery, The First Affiliated Hospital of Zhengzhou University, Zhengzhou, China; 2 Henan Liver Transplantation Centre, The First Affiliated Hospital of Zhengzhou University, Zhengzhou, China; 3 Henan Organ Transplantation Quality Control Centre, The First Affiliated Hospital of Zhengzhou University, Zhengzhou, China; 4 Open and Key Laboratory for Hepatobiliary and Pancreatic Surgery and Digestive Organ Transplantation at Henan Province, The First Affiliated Hospital of Zhengzhou University, Zhengzhou, China

**Keywords:** Apoptosis, HIRI, Inflammatory Response, Rhbdf2, Ubiquitination

## Abstract

**Background & Aims:**

Hepatic ischemia-reperfusion injury (HIRI) is one of the common complications of liver transplantation. Rhomboid 5 homolog 2 (Rhbdf2) plays a crucial role in apoptosis, inflammation, and liver injury, but its role and regulatory mechanism in HIRI remain unclear. The aim of this study was to investigate the role of Rhbdf2 in HIRI and elucidate its molecular mechanism.

**Methods:**

Rhbdf2 expression levels were detected in pre-ischemia–reperfusion (Pre) and post-ischemia–reperfusion (Post) livers. Western blot analysis, flow cytometry, quantitative real-time polymerase chain reaction, and immunofluorescence staining were used to investigate the effects of Rhbdf2 on hepatic ischemia-reperfusion (HI/R). The potential molecular mechanisms of the effects of Rhbdf2 on HI/R were investigated by combining RNA sequencing and mass spectrometry analysis, as well as co-immunoprecipitation and in vitro ubiquitination assays.

**Results:**

The level of Rhbdf2 protein was significantly increased in HI/R. Overexpression of Rhbdf2 in mice exacerbated HI/R-induced liver injury, apoptosis, and the inflammatory response, whereas knockdown of Rhbdf2 produced the opposite results. Mechanistically, overexpression of Rhbdf2 promoted the phosphorylation of mitogen-activated protein kinase kinase kinase 7 (MAP3K7, also known as TAK1), thereby activating the JNK/p38 signaling pathway and ultimately exacerbating HIRI. Mass spectrometry analysis, co-immunoprecipitation, and in vitro ubiquitination assays revealed that the E3 ubiquitin ligase constitutive photomorphogenic 1 (Cop1) interacts with Rhbdf2 and mediates its degradation through K48-linked ubiquitination, thereby inhibiting the TAK1- JNK/p38 axis and reducing HIRI.

**Conclusions:**

This study revealed that Rhbdf2 exacerbates HIRI by activating the TAK1- JNK/p38 axis, whereas Cop1-mediated Rhbdf2 ubiquitination and degradation can significantly inhibit this process. These findings provide potential therapeutic targets and insights for the clinical treatment of HIRI.

## Graphical abstract

**Figure:**
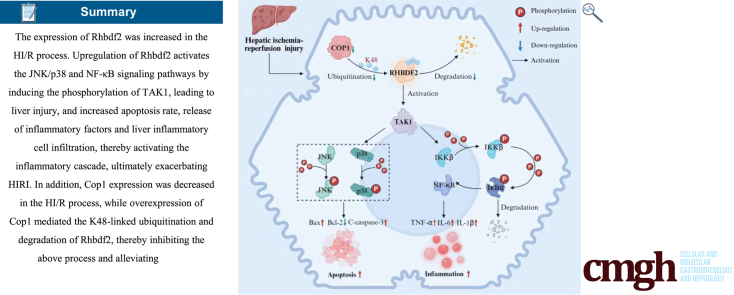

**Summary**

Our study revealed that rhomboid 5 homolog 2 aggravates hepatic ischemia-reperfusion injury by activating the transforming growth factor-β activating kinase 1-C-Jun N-terminal kinase/p38 signaling pathway, whereas constitutive photomorphogenic 1 mediated the ubiquitination and degradation of rhomboid 5 homolog 2, thereby inhibiting the transforming growth factor-β activating kinase 1-C-Jun N-terminal kinase/p38 axis and alleviating hepatic ischemia-reperfusion injury.

**What You Need to Know**

**Background**

Hepatic ischemia-reperfusion injury (HIRI) is a common surgical complication of liver transplantation; however, there is no effective treatment for HIRI. Therefore, it is crucial to explore its detailed molecular mechanisms.

**Impact**

This study found that constitutive photomorphogenic 1 (Cop1) alleviates HIRI by inhibiting the TAK1-JNK/p38 signal pathway through mediating the ubiquitination of Rhbdf2, which provides a new strategy for the clinical treatment of HIRI.

**Future Directions**

Based on the molecular mechanism of Cop1-mediated ubiquitination of Rhbdf2, to identify small molecule compounds that enhance Cop1 activity, thereby alleviating HIRI. This will provide novel insights for drug development to treat HIRI.

Liver transplantation is currently the primary clinical treatment for patients with end-stage liver disease, such as those with cirrhosis, autoimmune liver disease, drug-induced liver failure, and primary liver cancer. However, hepatic ischemia-reperfusion injury (HIRI) is an inevitable surgical complication of liver transplantation. This adverse event may lead to graft failure, liver dysfunction, and other adverse outcomes, significantly impacting patient survival rates.^1^ The mechanism of HIRI primarily involves 2 phases: ischemia and reperfusion. During the ischemia phase, the lack of blood supply leads to oxygen deprivation in hepatocytes, adenosine triphosphate (ATP) depletion, and cellular metabolic dysfunction. In the reperfusion phase, the restoration of blood supply triggers widespread activation of immune cells, release of proinflammatory factors, and accumulation of reactive oxygen species, which in turn activate an inflammatory cascade response, ultimately leading to HIRI.^2^, ^3^, ^4^, ^5^ However, there is currently no effective clinical treatment for HIRI. Therefore, elucidating its molecular mechanisms, identifying key regulatory targets, and developing strategies to effectively reduce hepatocyte damage and regulate inflammatory responses are of great theoretical and practical significance for improving the clinical prognosis of patients with HIRI.

Rhomboid 5 homolog 2 (Rhbdf2), also known as iRhom2, is a nonactive member of the rhombic membrane-associated protease family of serine proteases, which is involved in the regulation of a variety of biological processes.^6^^,^^7^ Current studies have indicated that overexpression of Rhbdf2 can activate inflammatory responses by promoting the transport and maturation of ADAM metallopeptidase domain 17 (ADAM17).^8^^,^^9^ Conversely, downregulation of Rhbdf2 expression can significantly reduce early atherosclerosis formation and inflammatory responses in Crohn’s disease.^10^^,^^11^ In liver diseases, inhibition of Rhbdf2 expression delays alcoholic liver fibrosis progression by reducing inflammatory responses and oxidative stress.^12^ In addition, Rhbdf2 promotes the phosphorylation of transforming growth factor-β activating kinase 1 (TAK1) and activates the nuclear factor kappa B (NF-κB) signaling pathway, driving the progression of nonalcoholic fatty liver disease (NAFLD).^13^ Although this biological process can be antagonized through the ubiquitination and degradation of Rhbdf2 by the E3 ubiquitin ligase tripartite motif containing 31 (Trim31).^14^ The detailed role of Rhbdf2 in hepatic ischemia/reperfusion (HI/R) and its molecular mechanisms remain unclear.

Constitutive photomorphogenic 1 (Cop1), also known as RNF200, is a highly conserved E3 ubiquitin ligase, which was initially identified in arabidopsis as a key regulator of photomorphogenesis.^15^^,^^16^ Subsequent studies have revealed that Cop1 plays a wide range of biological roles in mammals.^17^, ^18^, ^19^ Research has found that upregulating Cop1 can promote the ubiquitination and degradation of the transcription factor C/EBPβ, thereby attenuating neuroinflammatory responses, suggesting that Cop1 plays a crucial regulatory role in inflammatory diseases.^20^ However, studies on Cop1 in HIRI have not been reported to date. In this study, we found that Rhbdf2 expression was markedly increased in HI/R, and its increased expression level exacerbated liver damage, whereas inhibition of Rhbdf2 expression reduced the HI/R-induced inflammatory responses and apoptosis. Mechanistically, we found that Cop1 interacts with Rhbdf2 and mediates its K48-linked ubiquitination and degradation, thereby inhibiting the activation of the TAK1-C-Jun N-terminal kinase (JNK)/p38 axis and ultimately alleviating HIRI.

## Results

### Rhbdf2 Protein Expression Level Is Specifically Increased in HIRI

**Figure 1:**
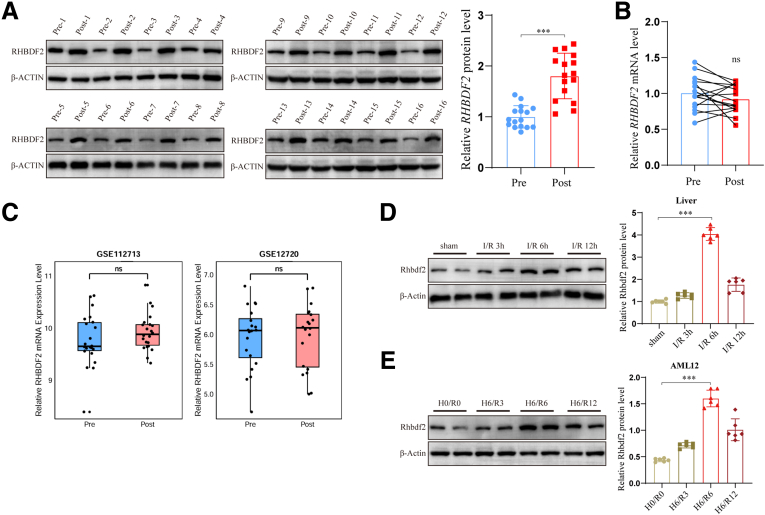
**The expression level of Rhbdf2 protein is significantly elevated in HIRI.** (*A*) Western blot analysis to determine RHBDF2 protein expression levels in Pre and Post livers of brain-dead donors (n = 16) and its statistical analysis. (*B*) RT-PCR analysis of mRNA expression levels of *RHBDF2* in liver tissues of 16 pairs of hepatic tissue samples of the Pre and Post groups (n = 16). (*C*) Analysis of *RHBDF2* mRNA expression levels in the GSE112713 and GSE12720 datasets. (*D*) Western blot analysis and quantification of hepatic Rhbdf2 protein in mice in the sham group and after ischemic 60-minute reperfusion for 3, 6, and 12 hours (n = 3 per group). (*E*) Western blot analysis and quantification of Rhbdf2 protein expression levels in AML12 cells from the control group and groups subjected to 6 hours of hypoxia followed by 3, 6, and 12 hours of reoxygenation (H6/R3, H6/R6, H6/R12) (n = 3 per group). Using β-Actin as loading control, data are shown as the mean ± SD; ns indicates no statistical difference compared with control; ∗*P* < .05; ∗∗*P* < .01; and ∗∗∗*P* < .001 indicate a statistical difference compared with control.

Current research indicates that Rhbdf2 plays a crucial regulatory role in a variety of diseases, such as cardiovascular disease, obesity, and rheumatoid arthritis.^21^^,^^22^ However, the specific role of Rhbdf2 in HIRI still unclear. In this study, the role of Rhbdf2 in HIRI was investigated in 16 pairs of pre-ischemia–reperfusion (Pre) and post-ischemia-reperfusion (Post) hepatic tissue samples collected from brain-dead donors. Western blot analysis results showed that Rhbdf2 protein expression levels were clearly higher in the Post group compared with the Pre group (Figure 1*A*). Notably, both the quantitative real-time polymerase chain reaction (qRT-PCR) test results and the bioinformatics analysis of the GSE112713 and GSE12720 Gene Expression Omnibus (GEO) datasets revealed that there was no significant statistical difference in the mRNA expression levels of Rhbdf2 between the Pre group and the Post group (Figure 1*B* and *C*). The above results suggest that post-translational modification of Rhbdf2 may be involved in the regulation of the HI/R process. In addition, we constructed an HI/R mouse model to further study the changes in Rhbdf2 in HIRI. Western blot analysis results revealed that, compared with the sham group, the HIRI group showed the highest Rhbdf2 protein expression level at 6 hours after reperfusion (Figure 1*D*). After subjecting AML12 cells to hypoxia-reoxygenation (H/R) exposure, the protein expression level of Rhbdf2 was also significantly increased after 6 hours of reoxygenation (Figure 1*E*). This is highly consistent with in vivo experimental results. These results indicate that Rhbdf2 is characteristically highly expressed in HIRI, and this upregulation primarily occurs at the protein level rather than the transcriptional level. This finding suggests that Rhbdf2 may regulate the pathological process of HI/R through specific molecular mechanisms, laying an important foundation for further in-depth research into its mechanism of action.

### Rhbdf2 Overexpression Exacerbates H/R-induced Hepatocyte Injury by Promoting Apoptosis and Inflammatory Response

**Figure 2:**
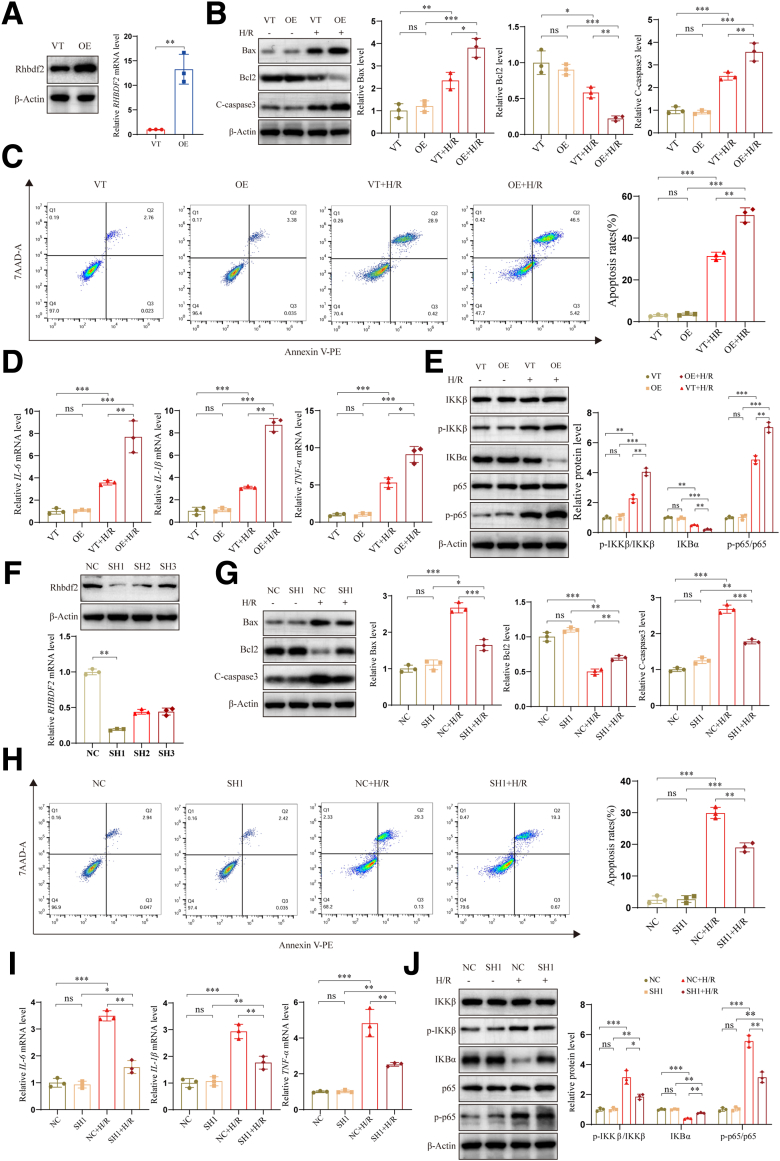
**Overexpression of Rhbdf2 exacerbated H/R-induced apoptosis and inflammatory response, whereas downregulation of Rhbdf2 significantly alleviates H/R-induced liver injury.** (*A*) Analysis of Rhbdf2 protein and mRNA expression levels in Rhbdf2 overexpressing cell lines (OE) and vector control cell lines (VT) by Western blot and qRT-PCR analyses (n = 3 per group). (*B*) Western blot analysis of the protein expression levels and quantification of Bax, Bcl2, and C-caspase 3 in the OE group and the VT group before and after H/R (n = 3 per group). (*C*) Flow cytometry analysis of apoptosis rate and statistical analysis of cells in the OE and VT groups after H/R (n = 3 per group). (*D*) qRT-PCR analysis of mRNA levels of *IL-6, IL-1β,* and *TNF-α* in the OE and VT groups after H/R (n = 3 per group). (*E*) Western blot analysis and quantification of the expression levels of NF-κB signaling pathway-related proteins in cells from the OE and VT groups before and after H/R (n = 3 per group). (*F*) Western blot analysis and qRT-PCR analysis to determine the protein and mRNA expression levels of Rhbdf2 respectively in Rhbdf2 knockdown cells (SH1, 2, 3) and control cells (NC) (n = 3 per group). (*G*) Western blot analysis of the protein expression levels of Bax, Bcl2, and C-caspase3 in the cells of the SH1 and NC groups before and after H/R, and the results of statistical analysis (n = 3 per group). (*H*) Flow cytometry analysis of apoptosis rate of SH1 cells and NC cells before and after H/R, and statistical analysis (n = 3 per group). (*I*) qRT-PCR analysis of mRNA expression levels of inflammatory cytokines *IL-6*, *IL-1β,* and *TNF-α* in SH1 and NC cells before and after H/R (n = 3 per group). (*J*) The expression levels and statistical analysis of NF-κB signaling pathway-related proteins in SH1 and NC cells before and after H/R (n = 3 per group). Using β-Actin as loading control; data are shown as the mean ± SD; ns indicates no statistical difference compared with control; ∗*P* < .05; ∗∗*P* < .01; ∗∗∗*P* < .001 indicate a statistical difference compared with control.

To investigate whether Rhbdf2 exacerbates apoptosis and inflammatory responses during the HIRI process, we established a Rhbdf2 overexpressing AML12 cell line (OE group) and vector control cell line (VT group) (Figure 2*A*). Western blot analysis results showed that after H/R exposure, the protein expression levels of apoptosis-related proteins BCL2-associated X protein (Bax) and cleaved caspase 3 (C-caspase3) were clearly higher in the OE group than in the VT group, whereas the protein expression levels of the anti-apoptotic protein B-cell lymphoma 2 (Bcl2) was noticeably lower (Figure 2*B*). Additionally, the detection of the apoptosis rate by flow cytometry indicated that the apoptosis rate in the VT group was markedly lower than that in the OE group after H/R exposure (Figure 2*C*). This finding indicated that Rhbdf2 overexpression significantly increased H/R-induced apoptosis. Additionally, qRT-PCR analysis results indicated that mRNA expression levels of the inflammatory factors interleukin-6 (IL-6), interleukin-1β (*IL-1β*), and tumor necrosis factor-α (*TNF-α*) were obviously increased in the OE group after H/R (Figure 2*D*). We also found that Rhbdf2 overexpression significantly activated the NF-κB signaling pathway, as indicated by increased protein phosphorylation levels of inhibitor of kappa B kinase (IKKβ) and p65, and decreased protein expression levels of inhibitor of kappa B alpha (IKBα) (Figure 2*E*). The Rhbdf2-induced activation of the NF-κB signaling pathway may be critical for its proinflammatory actions. To further validate the function of Rhbdf2, we established short hairpin RNA (shRNA)-mediated Rhbdf2 knockdown cell lines (SH groups) in AML12 cells and a control cell line (NC group). The results of the Western blot and qRT-PCR analyses showed that the SH1 group had the highest Rhbdf2 knockdown efficiency (Figure 2*F*). Therefore, the SH1 group was used as the Rhbdf2 knockdown cell line in subsequent experiments. The experimental results showed that inhibition of Rhbdf2 expression significantly reduced H/R-induced hepatocyte apoptosis and inflammatory response (Figure 2*G* and *H*). It also reduced the expression of inflammatory cytokines (Figure 2*I*). In addition, it also significantly inhibited the activation of the NF-κB signaling pathway (Figure 2*J*). This finding was in stark contrast to that of the Rhbdf2 overexpressing group after being subjected to H/R exposure. In summary, in vitro experiments indicated that overexpression of Rhbdf2 exacerbates hepatocyte apoptosis and inflammation during the HI/R process by promoting the expression of apoptosis-related proteins and activating the NF-κB signaling pathway. In contrast, inhibition of Rhbdf2 expression significantly alleviates HIRI.

### Rhbdf2 Overexpression Exacerbates I/R-induced Liver Injury by Promoting Apoptosis and Inflammation

**Figure 3:**
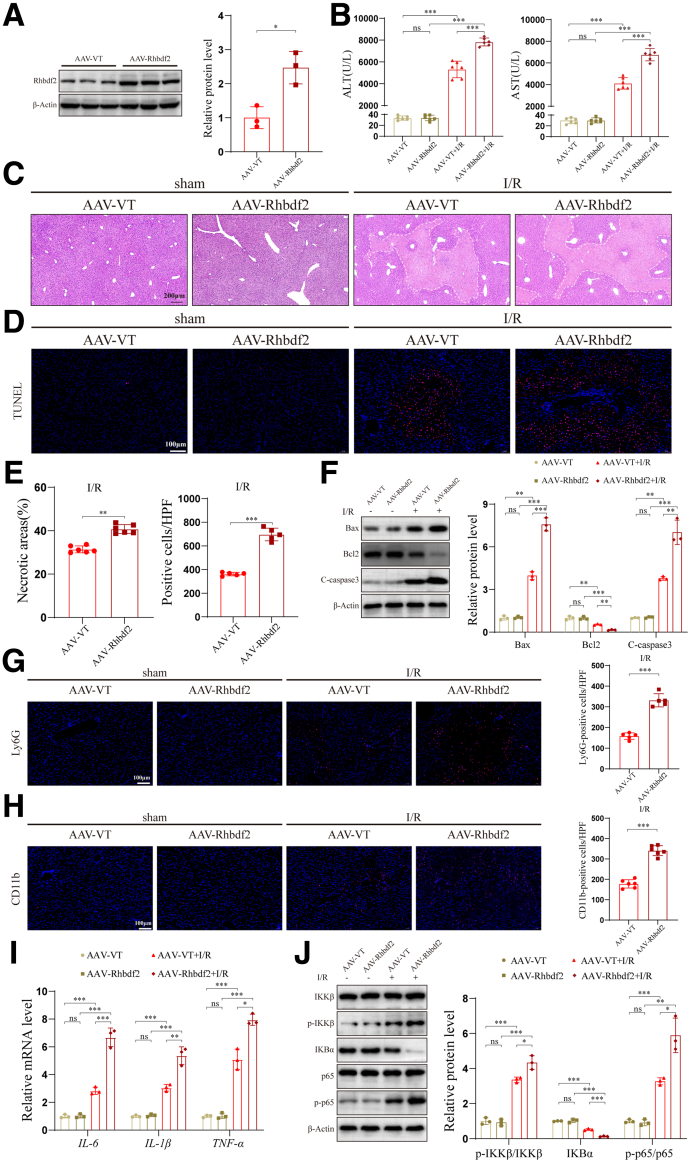
**Overexpression of Rhbdf2 exacerbated HI/R-induced liver injury, apoptosis, and inflammation.** (*A*) Western blot analysis of Rhbdf2 protein expression levels in Rhbdf2 overexpressing mice (AAV-Rhbdf2) and vector control mice (AAV-VT) (n = 3 per group). (*B*) ALT and AST levels in mice of the AAV-Rhbdf2 group and AAV-VT group after I/R 6 hours (n = 6 in each group). (*C–E*) H&E staining (scale bar, 200 μm), TUNEL staining (scale bar, 100 μm), and correspondent statistical analysis of the livers of mice in the AAV-Rhbdf2 group and the AAV-VT group after undergoing sham operation and I/R 6 hours (n = 6 per group). (*F*) Western blot analysis of the expression levels of Bax, Bcl2, and C-caspase 3 proteins and their quantification in the livers of mice after undergoing sham operation and I/R 6 hours (n = 3 per group). (*G* and *H*) Liver Ly6G, CD11b staining, and statistical analysis (scale bar, 100 μm) of mice from the AAV-Rhbdf2 group and AAV-VT group before and after I/R 6 hours (n = 6 per group). (*I*) qRT-PCR analysis to determine the mRNA expression levels of inflammatory cytokines *IL-6, IL-1β,* and *TNF-α* in the livers of the indicated groups after I/R 6 hours (n = 3 per group). (*J*) Western blot analysis and quantification of the expression levels of NF-κB signaling pathway-related proteins in the livers of mice undergoing the sham operation and after I/R 6 hours (n = 3 per group). Using β-Actin as loading control; data are shown as the mean ± SD; ns indicates no statistical difference compared with control; ∗*P* < .05; ∗∗*P* < .01; ∗∗∗*P* < .001 indicate a statistical difference compared with control.

Building on our previous in vitro finding that Rhbdf2 can exacerbate H/R-induced liver damage, we investigated whether Rhbdf2 performs the same function in vivo. To this end, we constructed Rhbdf2 overexpressing mice (AAV-Rhbdf2 group) and their corresponding vector control mice (AAV-VT group) through tail vein injection of the recombinant adeno-associated virus (AAV) expressing *Rhbdf2* or the AAV vector control (Figure 3*A*). After establishing the HI/R mouse model, we collected serum samples from the two groups of mice to measure their transaminase levels (alanine aminotransferase [ALT] and aspartate aminotransferase [AST]). The serological test results revealed that the ALT and AST levels were significantly lower in the AAV-VT group mice than in the AAV-Rhbdf2 group mice after HI/R (Figure 3*B*). Further histopathological analysis of liver tissue revealed that the areas of liver necrosis in the AAV-Rhbdf2 group mice were significantly larger than that in the AAV-VT group mice (Figure 3*C* and *E*). These results indicated that overexpression of Rhbdf2 exacerbated liver damage, as evidenced by elevated transaminase levels and increased areas of liver necrosis. To further investigate the effect of Rhbdf2 on hepatocyte apoptosis, we performed terminal deoxynucleotidyl-transferase dUTP nick-end labeling (TUNEL) staining analysis on paraffin embedded liver tissue sections. The results revealed a significant increase in the number of apoptotic cells in the liver tissue of AAV-Rhbdf2 treated mice (Figure 3*D* and *E*). This higher number of apoptotic cells is due to the significant increase in the protein expression levels of pro-apoptotic proteins Bax and C-caspase 3 in the liver following Rhbdf2 overexpression, as well as the decrease in the expression of the anti-apoptotic protein Bcl2 (Figure 3*F*). Sterile inflammatory responses played an important role in the HI/R process.^23^ Thus, we investigated whether Rhbdf2 affects inflammatory cell infiltration in the liver using immunofluorescence staining, which showed that Ly6G^+^ cells and CD11b^+^ cells were significantly increased in the livers of AAV-Rhbdf2 group mice (Figure 3*G* and *H*). In addition, qRT-PCR analysis results showed that the mRNA expression levels of inflammatory cytokines *IL-6*, *IL-1β*, and *TNF-α* were markedly increased in the liver tissue of the AAV-Rhbdf2 group mice (Figure 3*I*). Moreover, Western blot analysis results showed that overexpression of Rhbdf2 significantly activated the NF-κB signaling pathway, as indicated by increased levels of phosphorylated (p)-IKKβ and p-p65, and decreased expression of IKBα protein (Figure 3*J*). Consistent with the results of the in vitro experiments, the in vivo experiments also showed that Rhbdf2 overexpression markedly exacerbated HIRI by promoting hepatocyte apoptosis and activating NF-κB-mediated inflammatory responses.

### Rhbdf2 Downregulation Alleviates I/R-induced Liver Injury, Apoptosis, and Inflammation

**Figure 4:**
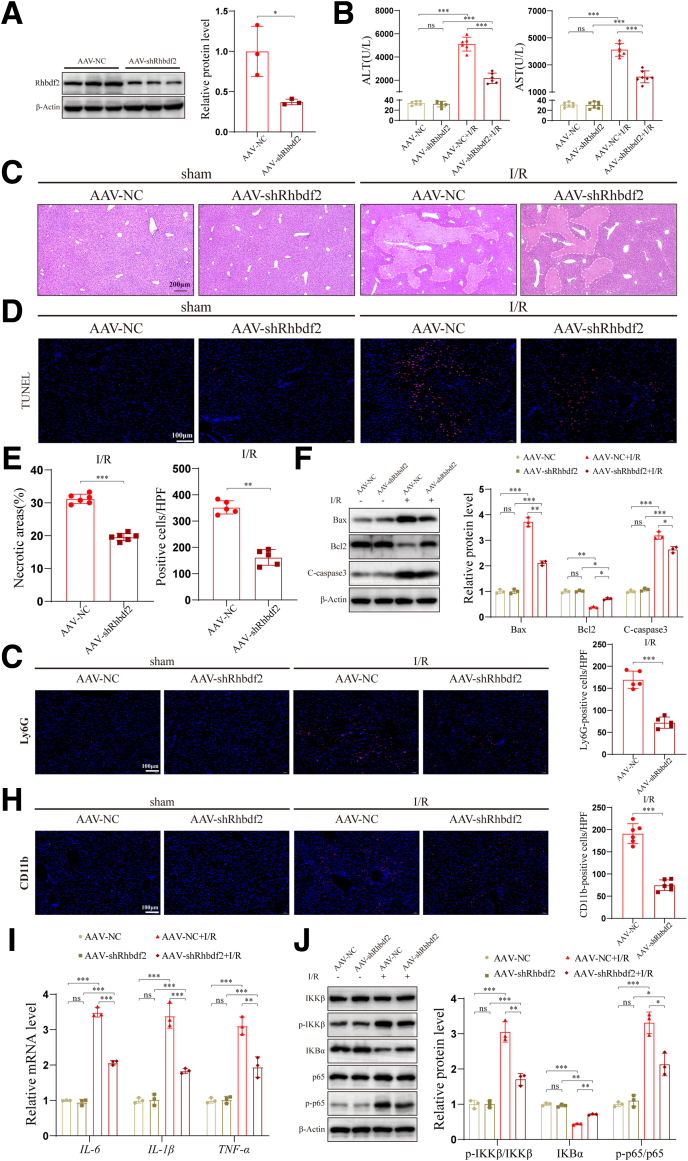
**Inhibition of Rhbdf2 reduced HI/R-induced liver injury, apoptosis, and inflammation.** (*A*) Western blot analysis of Rhbdf2 protein expression levels in Rhbdf2 knockdown mice (AAV-shRhbdf2) and control mice (AAV-NC) (n = 3 per group). (*B*) ALT and AST levels in mice in the AAV-shRhbdf2 group and AAV-NC group after I/R 6 hours (n = 6 per group). (*C–E*) H&E staining (scale bar, 200 μm), TUNEL staining (scale bar, 100 μm), and statistical analysis of the livers from mice of the AAV-shRhbdf2 group and AAV-NC group after undergoing sham operation and after I/R 6 hours (n = 6 per group). (*F*) Western blot analysis of the expression levels of the Bax, Bcl2, and C-caspase 3 proteins and their quantification in the livers of mice after undergoing sham operation and after I/R 6 hours (n = 3 per group). (*G* and *H*) Liver Ly6G, CD11b staining, and statistical analysis (scale bar, 100 μm) of mice in the AAV-shRhbdf2 group and AAV-NC group after undergoing sham operation and after I/R 6 hours (n = 6 per group). (*I*) qRT-PCR analysis of the mRNA expression levels of inflammatory cytokines *IL-6, IL-1β,* and *TNF-α* in the livers of the indicated groups after I/R (n = 3 per group). (*J*) Western blot analysis and quantification of the expression levels of NF-κB signaling pathway-related proteins in the livers of mice after undergoing sham operation and I/R 6 hours (n = 3 per group). Using β-Actin as loading control; data are shown as the mean ± SD; ns indicates no statistical difference compared with control; ∗*P* < .05; ∗∗*P* < .01; ∗∗∗*P* < .001 indicate a statistical difference compared with control.

It has been reported that inhibiting Rhbdf2 expression can reduce inflammatory responses and alleviate liver damage.^10^^,^^12^^,^^24^ Thus, we constructed an HI/R model to investigate whether inhibiting Rhbdf2 expression alleviates HIRI. To this end, we performed *Rhbdf2* silencing in HI/R model mice using a *Rhbdf2* silencing AAV construct (AAV-shRhbdf2) expressing shRNA targeting Rhbdf2 expression and a control construct (AAV-NC) expressing a scrambled shRNA (Figure 4*A*). Serological tests showed that, compared with the AAV-NC group mice, the serum levels of ALT and AST in the AAV-shRhbdf2 group mice were significantly reduced (Figure 4*B*). In addition, hematoxylin and eosin (H&E) staining of the liver tissues of the two groups of mice showed significantly reduced necrotic area in the liver of the Rhbdf2 silenced mice (Figure 4*C* and *E*). These findings indicate that inhibiting the expression of Rhbdf2 can significantly reduce HI/R-induced liver damage. Additionally, TUNEL staining results revealed that inhibiting Rhbdf2 expression significantly inhibited hepatocyte apoptosis (Figure 4*D* and *E*). This was due to the marked reduction of the protein expression levels of pro-apoptotic proteins Bax and C-caspase 3, and the increase of the expression of the anti-apoptotic protein Bcl2 following the inhibition of Rhbdf2 expression (Figure 4*F*), leading to the inhibition of the HI/R-induced apoptotic process in hepatocytes. However, in contrast to the immunofluorescence staining results of Rhbdf2 overexpressing mice, the AAV-shRhbdf2 group mice showed a significant reduction in Ly6G^+^ and CD11b^+^ cell infiltration in the liver (Figure 4*G* and *H*). Also, qRT-PCR analysis results showed that the mRNA expression levels of inflammatory cytokines *IL-6*, *IL-1β,* and *TNF*-α were significantly reduced (Figure 4*I*). In addition, Western blot analysis results revealed that the inhibition of Rhbdf2 expression significantly reduced the activation of the NF-κB signaling pathway during the HI/R process (Figure 4*J*). In conclusion, taken together, the above results demonstrate that Rhbdf2 gene silencing in HI/R model mice by shRNA expression using an AAV construct can reduce the area of liver necrosis and inhibit liver inflammatory cell infiltration, thereby alleviating HIRI.

### Overexpression of Rhbdf2 Can Reverse the Protective Effect of the Downregulation of Rhbdf2 on H/R

**Figure 5:**
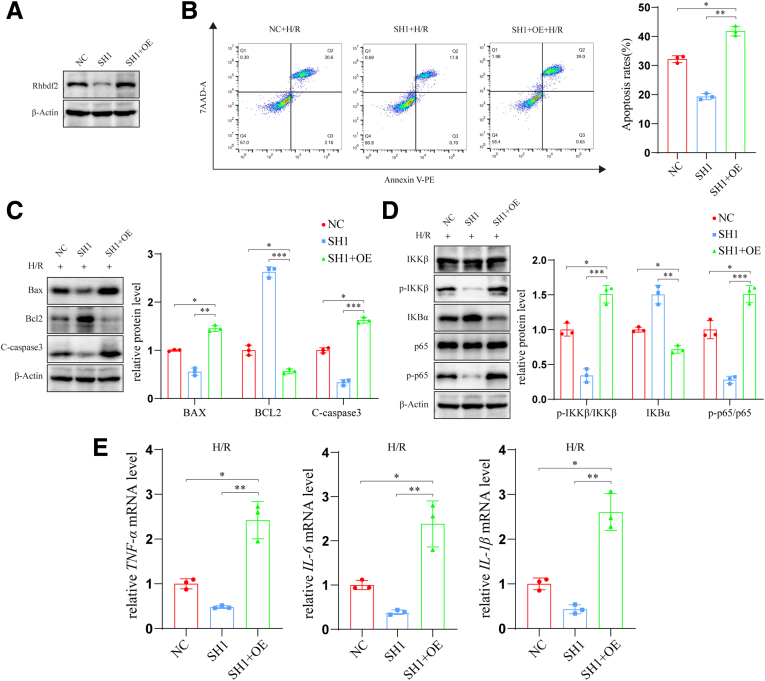
**Overexpression of Rhbdf2 can reverse the protective effect of the downregulation of Rhbdf2 on H/R.** (*A*) The expression of Rhbdf2 protein in the NC group, SH1 group and SH1+OE group (n = 3 per group). (*B*) Flow cytometry analysis of apoptosis rate and statistical analysis of cells in the NC group, SH1 group, and SH1+OE group after H/R (n = 3 per group). (*C*) Western blot analysis of the protein expression levels and quantification of Bax, Bcl2, and C-caspase 3 in NC group, SH1 group, and SH1+OE group after H/R (n = 3 per group). (*D*) Western blot analysis and quantification of the expression levels of NF-κB signaling pathway-related proteins in NC group, SH1 group, and SH1+OE group after H/R (n = 3 per group). (*E*) qRT-PCR analysis of mRNA expression levels of inflammatory cytokines *IL-6*, *IL-1β,* and *TNF-α* in NC group, SH1 group, and SH1+OE group after H/R (n = 3 per group). Using β-Actin as loading control; data are shown as the mean ± SD; ns indicates no statistical difference compared with control; ∗*P* < .05; ∗∗*P* < .01; ∗∗∗*P* < .001 indicate a statistical difference compared with control.

Our previous experiments showed that Rhbdf2 promotes liver injury, cell apoptosis, and inflammatory responses in HIRI. To further confirm its role and rule out nonspecific interference, we transfected Rhbdf2 overexpression plasmids into Rhbdf2-knockdown AML12 cell lines (designated as the SH1+OE group). This group was then compared with Rhbdf2-knockdown cells (SH1 group) and control cells (NC group) (Figure 5*A*). Flow cytometry analysis revealed that overexpression of Rhbdf2 significantly reversed the inhibitory effect of Rhbdf2 knockdown on H/R-induced cell apoptosis after H/R (Figure 5*B*). In addition, Western blot analysis results showed that the expression levels of the apoptosis-related proteins Bax and C-caspase3 in the SH1+OE group were significantly increased compared with the SH1 group, whereas the expression of anti-apoptotic protein Bcl2 was significantly reduced (Figure 5*C*). Additionally, we verified the expression of proteins associated with the NF-κB signaling pathway. The results showed that overexpression of Rhbdf2 significantly reversed the inhibitory effect of Rhbdf2 knockdown on the NF-κB signaling pathway (Figure 5*D*). Finally, after overexpression of Rhbdf2, the mRNA levels of the inflammatory factors *IL-6*, *IL-1β*, and *TNF-α* were also significantly higher than those of the SH1 group. These results are consistent with the previous findings, further confirming that Rhbdf2 plays a role in promoting apoptosis and inflammatory response in HIRI.

### Rhbdf2 Promotes HIRI Through the TAK1- JNK/p38 Axis

**Figure 6:**
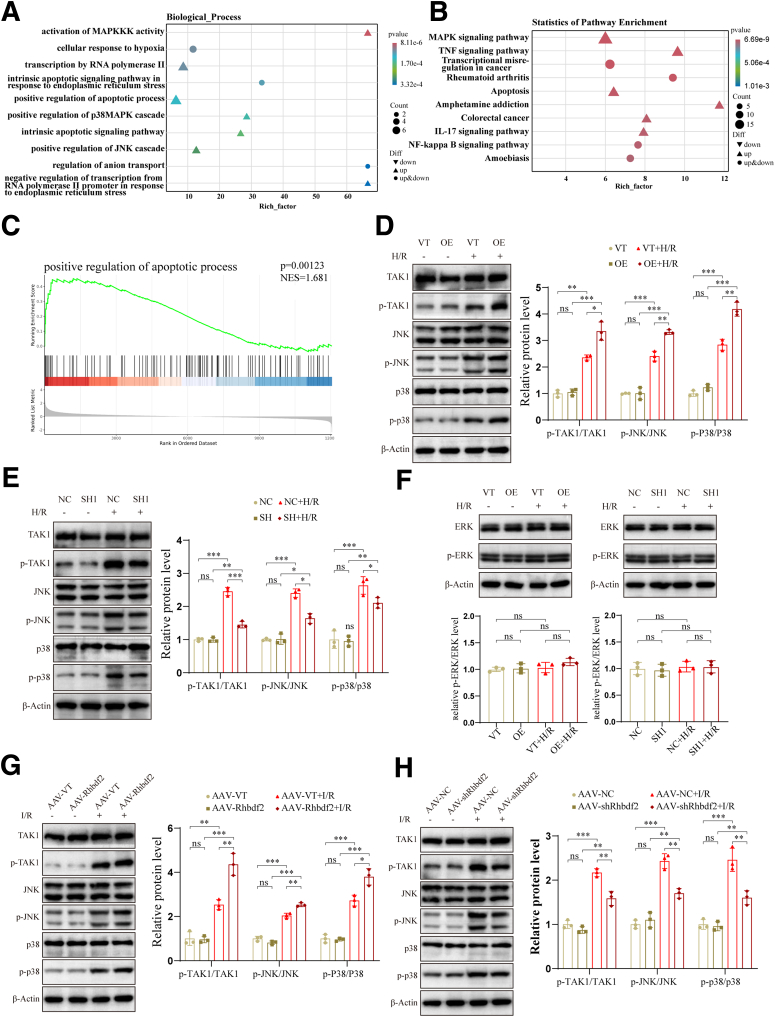
**Rhbdf2 promotes HIRI by activating the TAK1-JNK/p38 axis.** (*A*) GO term enrichment analysis of RNA-seq data showing significantly enriched function in the control and Rhbdf2 knockdown cells after H/R (n = 5 per group). (*B*) KEGG pathway enrichment analysis of RNA-seq data showing significant enrichment of the pathways after H/R in control and Rhbdf2 knockdown cells (n = 5 per group). (*C*) GSEA of RNA-Seq data from control and Rhbdf2 knockdown cells after H/R (n = 5 per group). (*D*) Western blot analysis and quantification of the expression levels of TAK1, JNK, and p38 proteins and corresponding phosphorylated proteins in OE and VT cells before and after H/R (n = 3 per group). (*E*) Western blot analysis and quantification of the expression levels of TAK1, JNK, and p38 proteins and corresponding phosphorylated proteins in SH1 and NC cells before and after H/R (n = 3 per group). (*F*) Western blot analysis and quantification of ERK and p-ERK protein expression levels in VT/OE cell lines and NC/SH1 cell lines before and after H/R (n = 3 per group). (*G*) Western blot analysis and quantification of the expression levels of TAK1, JNK, and p38 proteins and corresponding phosphorylated proteins in livers of AAV-Rhbdf2 and AAV-VT mice after undergoing sham operation and I/R 6 hours (n = 3 per group). (*H*) Western blot analysis and quantification of the expression levels of TAK1, JNK, and p38 proteins and corresponding phosphorylated proteins in the livers of AAV-shRhbdf2 and AAV-NC mice after undergoing sham operation and I/R 6 hours (n = 3 per group). Using β-Actin as loading control; data are shown as the mean ± SD; ns indicates no statistical difference compared with corresponding control; ∗*P* < .05; ∗∗*P* < .01; ∗∗∗*P* < .001 indicate a statistical difference compared with corresponding control.

Previous studies have shown that Rhbdf2 binds to TAK1 and promotes its phosphorylation. Increased p-TAK1 activates JNK phosphorylation and the NF-κB signaling pathway, thereby exacerbating NAFLD.^13^ However, the specific molecular mechanism of Rhbdf2 in HIRI remains unclear. Therefore, to investigate the mechanism by which Rhbdf2 exacerbates HIRI, we performed RNA sequencing (RNA-seq) analysis on RNA extracted from Rhbdf2 knockdown cell lines and their control groups after being subjected to H/R exposure. Gene Ontology (GO) term enrichment analysis of differentially expressed genes (DEGs) indicated that Rhbdf2 promoted the activation of MAPKKKs, and is involved in regulating the activation of the p38 and JNK signaling pathways (Figure 6*A*). Kyoto Encyclopedia of Genes and Genomes (KEGG) pathway enrichment analysis results showed that upregulation of Rhbdf2 led to activation of the mitogen-activated protein kinase (MAPK), apoptosis, and NF-κB signaling pathways (Figure 6*B*). Additionally, gene set enrichment analysis (GSEA) results revealed that changes in Rhbdf2 are closely associated with apoptosis (Figure 6*C*). Studies on NAFLD have shown that Rhbdf2 interacts with TAK1 to promote its phosphorylation. As one of the crucial members of the MAPKKK family, p-TAK1 can activate the MAPK and NF-κB signaling pathways, thereby participating in the regulation of multiple biological processes, including cellular damage and inflammatory responses in various diseases.^25^, ^26^, ^27^ In this study, the Western blot analysis results revealed that overexpression of Rhbdf2 significantly promoted TAK1 phosphorylation, and that p-TAK1 activated the JNK/p38 signaling pathway, resulting in significantly increased protein expression levels of p-JNK and p-p38, but there were no significant changes in TAK1, JNK, and p38 protein levels (Figure 6*D*). In contrast to the above results, inhibition of Rhbdf2 expression significantly suppressed the TAK1-JNK/p38 axis (Figure 6*E*). However, there were no significant changes in the protein levels of ERK and p-ERK (Figure 6*F*). These findings indicate that during the HI/R process, Rhbdf2 promotes the phosphorylation of TAK1, and p-TAK1 activates the JNK/p38 signaling pathway, thereby exacerbating HIRI. This conclusion was also confirmed in Rhbdf2 transgenic mice (Figure 6*G* and *H*). In summary, increased expression of Rhbdf2 leads to the accumulation of p-TAK1, which in turn activates the JNK/p38 signaling pathway, ultimately resulting in aggravated liver damage, increased cell apoptosis, and enhanced inflammatory responses. Therefore, inhibiting the expression of Rhbdf2 can alleviate the pathological progression of HI/R.

### Cop1 Alleviates HIRI and Mediates Ubiquitination of Rhbdf2

**Figure 7:**
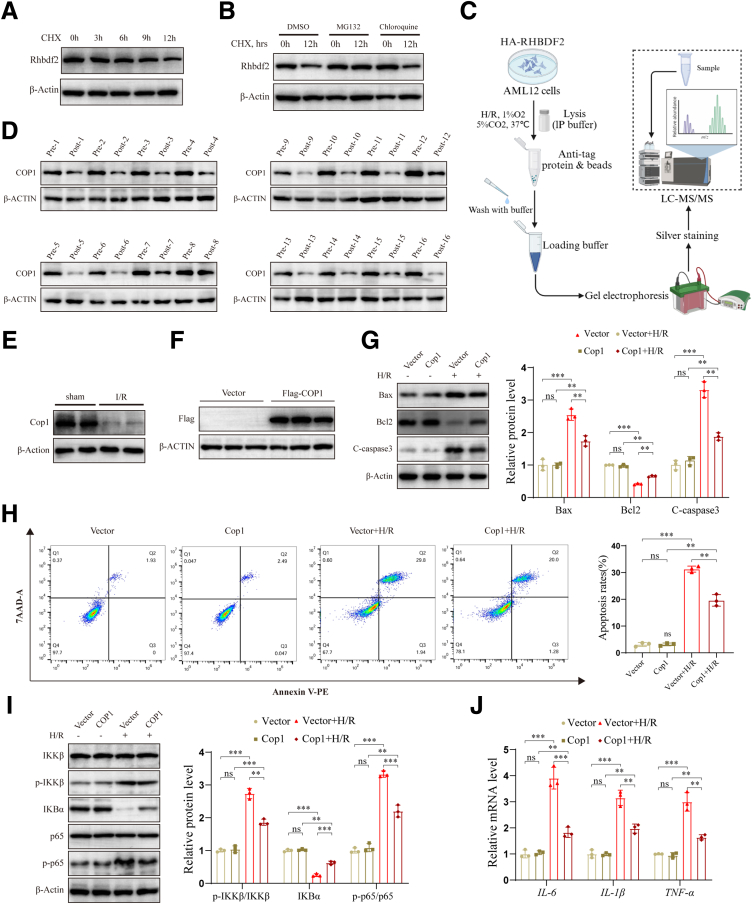
**Mass spectrometry analysis found that Rhbdf2 interacts with Cop1, and upregulating Cop1 can significantly alleviate HIRI.** (*A*) Western blot analysis of Rhbdf2 protein expression levels in AML12 cells after treatment with CHX (50 μg/mL) for 0, 3, 6, 9, and 12 hours, respectively (n = 3 per group). (*B*) Western blot analysis of Rhbdf2 protein expression levels in AML12 cells after treatment with CHX following the addition of DMSO (at 0 and 12 hours), MG132 (25 μM; at 0 and 12 hours), and chloroquine (50 μM, at 0 and 12 hours), respectively (n = 3 per group). (*C*) Mass spectrometry analysis (using LC-MS/MS) of Rhbdf2 binding proteins in Rhbdf2 overexpressing cells and control cells after H/R. (*D*) Western blot analysis to determine Cop1 protein expression levels in Pre and Post livers of brain-dead donors (n = 16). (*E*) Western blot analysis of protein expression levels of Cop1 in the livers of mice undergoing sham operation or I/R 6 hours (n = 3 per group), and statistical analysis. (*F*) Western blot analysis of Cop1 protein expression levels in Cop1 overexpressing cells (Flag-COP1) and control cells (Vector) (n = 3 per group). (*G*) Western blot analysis of Bax, Bcl2, and C-caspase 3 protein expression levels in the Cop1 and Vector groups before and after H/R, and statistical analysis (n = 3 per group). (*H*) Flow cytometry analysis of the cell apoptosis rates in the Cop1 and Vector groups before and after H/R. Statistical analysis (n = 3 per group). (*I*) Western blot analysis of the expression levels of the IKKβ, IKBα, and p65 proteins and their phosphorylated proteins in the Cop1 and Vector groups before and after H/R. Statistical analysis (n = 3 per group). (*J*) qRT-PCR analysis of mRNA expression levels of inflammatory cytokines in cells of the Cop1 and Vector groups before and after H/R (n = 3 per group). Using β-Actin as loading control; data are shown as the mean ± SD; ns indicates no statistical difference compared with control; ∗*P* < .05; ∗∗*P* < .01; ∗∗∗*P* < .001 indicate a statistical difference compared with control.

**Figure 8:**
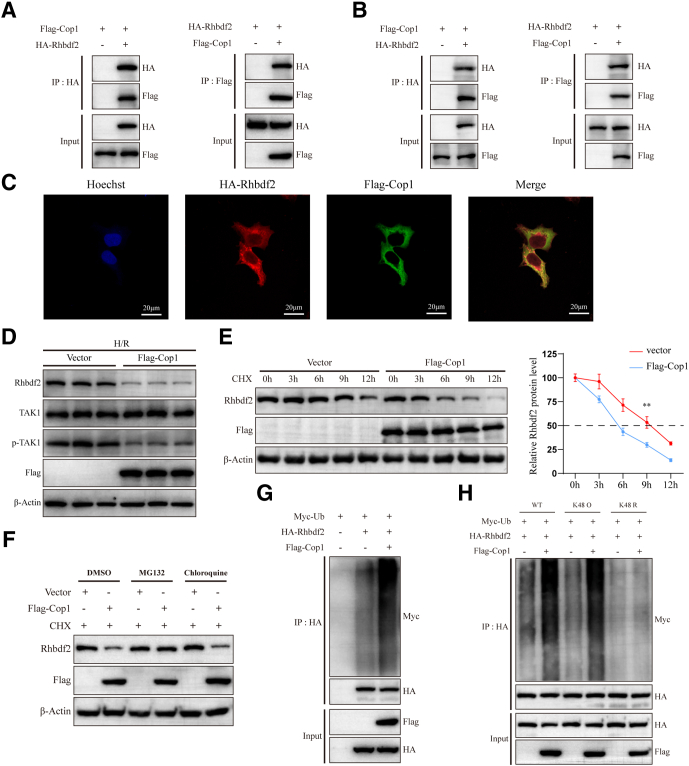
**Rhbdf2 binds to Cop1 and is regulated by Cop1-mediated ubiquitination and degradation.** (*A*) Co-transfection of plasmids expressing HA-Rhbdf2 and Flag-Cop1 into 293T cells and Co-IP analysis of the interaction between Rhbdf2 and Cop1 (n = 3 per group). (*B*) Co-transfection of HA-Rhbdf2-expressing plasmid and Flag-Cop1-expressing plasmid into AML12 cells and Co-IP analysis of the interaction between Rhbdf2 and Cop1 (n = 3 per group). (*C*) Confocal image showing co-localization of exogenous HA-Rhbdf2 (*red*) and Flag-Cop1 (*green*) in AML12 cells (n = 3 per group). (*D*) Western blot analysis of the expression levels of the Cop1, Rhbdf2, TAK1, and p-TAK1 proteins in Cop1 overexpressing (Flag-Cop1) and control (Vector) cell lines after H/R (n = 3 per group). (*E*) Western blot analysis and statistical analysis of Rhbdf2 and Cop1 protein expression levels in Cop1 overexpressing and control cell lines treated with CHX (50μg/mL) for 0, 3, 6, 9, and 12 hours, respectively (n = 3 per group). (*F*) Western blot analysis of Rhbdf2 and Cop1 proteins levels after addition of DMSO (0 and 6 hours), MG132 (25 μM, 0 and 6 hours), and chloroquine (50 μM, 0 and 6 hours) in Cop1 overexpressing and control cells after addition of CHX, respectively (n = 3 per group). (*G*) Western blot analysis of the effect of Cop1 on the level of ubiquitination of Rhbdf2 (n = 3 per group). (*H*) Western blot analysis of Rhbdf2 ubiquitination levels in AML12 cells co-transfected with Myc-Ub (WT, K48 O, K48 R) and HA-Rhbdf2 after transfection with Vector and Flag-Cop1 (n = 3 per group). Using β-Actin as loading control; data are shown as the mean ± SD; ns indicates no statistical difference compared with control; ∗*P* < .05; ∗∗*P* < .01; ∗∗∗*P* < .001 indicate a statistical difference compared with control.

Because Rhbdf2 protein levels significantly increased after HIRI, but mRNA expression did not change much, we hypothesized that Rhbdf2 regulates HIRI through post-translational modification. To verify this hypothesis, we first investigated the protein stability of Rhbdf2 by treating AML12 cells with cycloheximide (CHX, 50 μg/mL) at different time points to determine the half-life of Rhbdf2 protein degradation. Further treatment with the proteasome inhibitor MG132 (25 μM) and the lysosomal inhibitor chloroquine (50 μM) revealed that Rhbdf2 is primarily degraded via the proteasome pathway (Figure 7*A* and *B*). To identify molecules that interact and regulate Rhbdf2, we subjected Rhbdf2-overexpressing and control cell lines to H/R, we isolated Rhbdf2-interacting proteins by immunoprecipitation and identified them by mass spectrometry analysis (Figure 7*C*). The results revealed that Rhbdf2 interacts with the E3 ubiquitin ligase Cop1. Subsequent examination of the protein expression levels of Cop1 in 16 pairs of Pre and Post hepatic tissue samples collected from brain-dead donors showed that Cop1 protein expression levels were significantly lower in the Post group (Figure 7*D*). In addition, we found that, compared with the HI/R group, the sham group had significantly higher Cop1 expression levels (Figure 7*E*). We also examined the role of Cop1 in the HI/R process by generating a Cop1 overexpressing cell line (COP1 group) and a control cell line (Vector control group) for use in the analysis of the expression levels of impacted proteins (Figure 7*F*). Overexpression of Cop1 resulted in a decrease in the protein expression levels of pro-apoptotic proteins Bax and C-caspase3, a significant upregulation of the anti-apoptotic protein Bcl2, and a significantly lower rate of apoptosis rate compared with the Vector control group (Figure 7*G* and *H*). In addition, the expression levels of inflammatory cytokines was reduced in the Cop1 overexpression group, as a result of the Cop1 overexpression-induced inhibition of the activation of the NF-κB signaling pathway (Figure 7*I* and *J*). This finding indicated that Cop1 plays a protective role in the HI/R process, with biological functions antagonistic to those of Rhbdf2. Therefore, we hypothesized that Cop1 regulates Rhbdf2 to alleviate HIRI. We further verified the interaction between Cop1 and Rhbdf2 by co-immunoprecipitation (Co-IP) assays after co-transfecting HA-Rhbdf2 and Flag-Cop1 into 293T cells (Figure 8*A*). Additionally, we simultaneously transfected Cop1 and Rhbdf2 overexpression plasmids into AML12 cells, and the Co-IP results showed that Cop1 and Rhbdf2 also interacted in AML12 cells (Figure 8*B*). Furthermore, Rhbdf2 strongly co-localized with Cop1 (Figure 8*C*). Western blot analysis results showed that overexpression of Cop1 significantly reduced Rhbdf2 protein levels and inhibited TAK1 phosphorylation (Figure 8*D*), which confirmed our hypothesis. Because Cop1 is an E3 ubiquitin ligase, we further examined whether Cop1 mediated the ubiquitination and degradation of Rhbdf2 to exert its protective effect. The results showed that Cop1 overexpression significantly shortened the protein degradation half-life of Rhbdf2 (Figure 8*E*). This process by which Cop1 accelerates Rhbdf2 degradation was blocked by the proteasome inhibitor MG132 (Figure 8*F*). Together, these results indicated that Cop1 promotes the degradation of Rhbdf2 through the proteasome system. We also found, through in vitro ubiquitination experiments, that Cop1 can mediate Rhbdf2 K48-linked ubiquitination and degrade Rhbdf2 through the proteasome system (Figure 8*G* and *H*). Thus, our results indicate that Cop1 interacts with Rhbdf2 and mediates its K48-linked ubiquitination, thereby inhibiting TAK1 phosphorylation.

### Overexpression of Rhbdf2 Can Reduce the Protective Effect of Cop1 on HI/R

**Figure 9:**
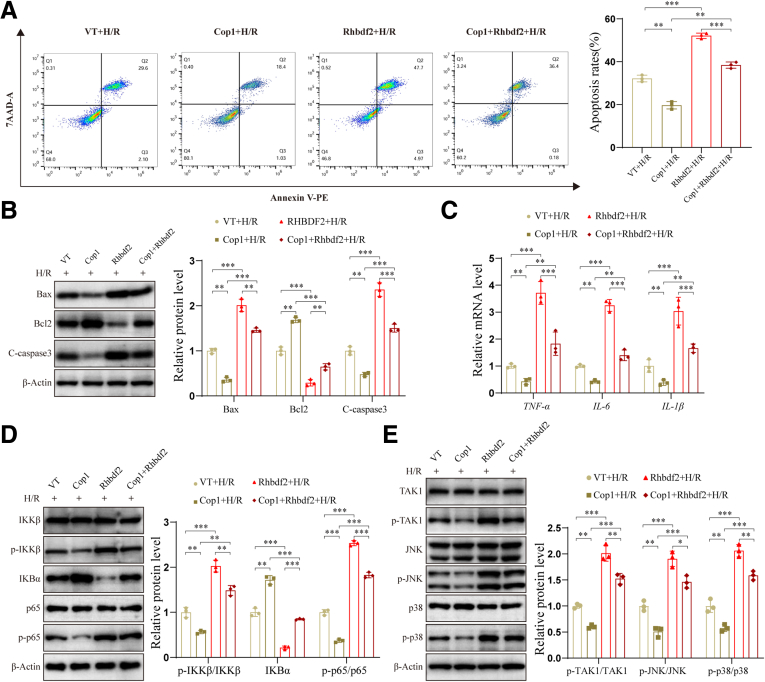
**Overexpression of Rhbdf2 inhibits the protective effect of Cop1.** (*A*) Plasmids carrying the indicated genes (VT, Cop1, Rhbdf2, Cop1+Rhbdf2) were transfected into AML12, and apoptotic rate of each group was determined by flow cytometry after H/R and statistical analysis (n = 3 per group). (*B*) Western blot analysis and statistical analysis of the expression levels of Bax, Bcl2, and C-caspase3 proteins in the indicated groups after H/R (n = 3 per group). (*C*) RT-PCR analysis of mRNA levels of *IL-6,IL-1β,* and *TNF-α* in the indicated groups after H/R (n = 3 per group). (*D*) Western blot analysis and quantitative analysis of the expression levels of NF-κB signaling pathway-associated proteins in each group after H/R (n = 3 per group). (*E*) Western blot analysis and quantification of the expression levels of TAK1, JNK, and p38 proteins and their phosphorylated proteins in each group after H/R (n = 3 per group). Using β-Actin as loading control; data are shown as the mean ± SD; ns indicates no statistical difference compared with control; ∗*P* < .05; ∗∗*P* < .01; ∗∗∗*P* < .001 indicate a statistical difference compared with control.

Our study indicates that Cop1 plays a protective role in HI/R and can mediate the K48-linked ubiquitination and degradation of Rhbdf2. Further rescue experiments were conducted to determine whether the protective role of Cop1 is achieved by mediating the ubiquitination and degradation of Rhbdf2. We co-transfected Cop1-overexpressing plasmids with Rhbdf2-overexpressing plasmids (Cop1+Rhbdf2) in AML12 cells to determine whether upregulating Rhbdf2 while overexpressing Cop1 could reduce the protective effect of Cop1. Flow cytometry analysis results indicated that, compared with the Cop1 overexpression group alone, the Cop1+Rhbdf2 group had significantly increased apoptotic rate, although it was still lower than that of the Rhbdf2 overexpression group alone (Figure 9*A*), which indicates that Rhbdf2 can partially reverse the protective effect of Cop1. This finding is further supported by the Western blot analysis results, which found higher protein expression levels of the pro-apoptotic proteins Bax and C-caspase3 in the Cop1+Rhbdf2 group than in the Cop1 group, and significantly reduced expression level of the anti-apoptotic protein Bcl2 (Figure 9*B*). These findings indicate that Rhbdf2 can reduce the protective effect of Cop1 on cells and rescue H/R-induced apoptosis. The results of the qRT-PCR and Western blot analyses showed that overexpression of Rhbdf2 could counteract the inhibition of the NF-κB signaling pathway by Cop1 and rescue the expression levels of inflammatory cytokines, thereby promoting H/R-induced inflammatory responses (Figure 9*C* and *D*). In addition, Rhbdf2 upregulation can rescue the inhibitory effect of Cop1 overexpression on the TAK1-JNK/p38 axis, as indicated by increased p-TAK1 protein levels and rescue of JNK/p38 activation. However, this rescue is limited compared with simply overexpressing Rhbdf2 (Figure 9*E*). Overall, these results indicate that Cop1 alleviates HIRI by mediating the ubiquitination and degradation of Rhbdf2, thereby inhibiting the TAK1- JNK/p38 axis, while overexpression of Rhbdf2 can partially reverse the protective effect of Cop1.

## Discussion

Liver transplantation is currently a common clinical treatment for end-stage liver disease, and HIRI is one of the main challenges facing liver transplantation today. It can lead to postoperative liver necrosis, liver dysfunction, and graft failure, seriously affecting patient prognosis.^2^ This study found that Rhbdf2 expression was significantly increased during the HI/R process, exacerbating liver injury, apoptosis, and inflammatory responses. It also elucidated the molecular mechanism by which Rhbdf2 regulates HIRI, providing new insights and a theoretical basis for the clinical treatment of HIRI.

Currently, the levels of transaminases, size of liver necrosis area, hepatocyte apoptosis count, and degree of inflammatory response are used to assess the severity of HIRI.^28^, ^29^, ^30^ This study revealed that the protein expression level of Rhbdf2 is increased in HI/R. In addition, overexpression of Rhbdf2 significantly exacerbated the HI/R-induced liver dysfunction, as indicated by the markedly elevated serum levels of ALT and AST. Some studies have found that Rhbdf2 can promote inflammatory responses through ADAM17.^8^^,^^31^ Moreover, Rhbdf2 is involved in the regulation of the NF-κB signaling pathway, and the activation of the NF-κB signaling pathway promotes the release of inflammatory cytokines and inflammatory cell infiltration, thereby activating the inflammatory cascade.^10^^,^^32^^,^^33^ Furthermore, upregulation of Rhbdf2 has been reported to promote inflammatory cell infiltration and liver damage.^12^^,^^34^ Accordingly, we investigated the relationship between Rhbdf2 and inflammatory responses and apoptosis in HIRI. The results showed that overexpression of Rhbdf2 activated the NF-κB signaling pathway, significantly increased the expression of inflammatory cytokines *TNF-α*, *IL-6*, and *IL-1β*, and led to inflammatory cell infiltration. The immunofluorescence staining of paraffin-embedded sections showed that the levels of neutrophil and macrophage infiltration in liver tissue were significantly higher in the Rhbdf2 overexpression group than in the control group. Additionally, our study found that the area of liver necrosis was significantly increased in the Rhbdf2 overexpression group, with a higher hepatocyte apoptotic rate than in the control group. The protein levels of the pro-apoptotic proteins Bax and C-caspase 3 were increased, whereas the level of the anti-apoptotic protein Bcl2 was decreased. In contrast, inhibition of Rhbdf2 expression resulted in the opposite effects, indicating that inhibiting Rhbdf2 expression can alleviate HIRI. These findings indicate that overexpression of Rhbdf2 induces inflammatory responses during the HI/R process, exacerbates liver damage, and promotes hepatocyte apoptosis.

Numerous studies have shown that the MAPK signaling pathway is activated during the HI/R process.^35^^,^^36^ TAK1 is one of the crucial members of the MAPKKK family, exacerbates liver injury, and promotes biological processes, such as inflammatory responses and apoptosis, by activating the MAPK signaling pathway.^26^^,^^37^ Additionally, current research has indicated that Rhbdf2 expression is increased in NAFLD, where it binds to TAK1 and promotes its phosphorylation, thereby activating the JNK signaling pathway and exacerbating liver damage and inflammatory responses.^13^ Our RNA-seq results further support these findings. GO term enrichment analysis and GSEA revealed that Rhbdf2 is strongly associated with MAPKKK activation, apoptosis, and inflammatory response in the HI/R process, whereas KEGG pathway enrichment analysis showed that the MAPK signaling pathway is closely related to Rhbdf2-exacerbated liver damage. Our results also indicated that the level of p-TAK1 was significantly increased after overexpression of Rhbdf2, and p-TAK1 activated the JNK/p38 signaling pathway, which ultimately led to overactivation of inflammatory responses and increased apoptosis. In contrast, inhibition of Rhbdf2 led to a significant reduction in the protein expression levels of p-TAK1, p-JNK, and p-p38. However, the phosphorylation level of ERK did not correlate with the changes of Rhbdf2. This finding revealed that the mechanism of Rhbdf2 in aggravating liver injury, promoting inflammatory response, and apoptosis involved the activation of the TAK1-JNK/p38 axis.

Cop1 is a member of the E3 ubiquitin ligase family and plays a key role in photomorphogenesis in plants. Recent studies have shown that Cop1 is involved in regulating inflammatory responses and tumorigenesis in animals.^19^^,^^20^^,^^38^^,^^39^ In this study, we found that Cop1 expression was reduced in the HI/R process, whereas Cop1 overexpression alleviated HIRI by attenuating the inflammatory response and inhibiting cell apoptosis. Our previous results showed that changes in Rhbdf2 in the HI/R process mainly occur at the protein levels, with no significant changes in the mRNA expression levels. Furthermore, other studies indicate that Rhbdf2 expression is regulated by ZDHHC3-mediated palmitoylation and TRIM31-mediated ubiquitination.^14^^,^^40^ Therefore, we hypothesized that Rhbdf2 exerts its biological effects through post-translational modifications during the HI/R process. Our study found that Rhbdf2 can be degraded by the proteasome. Moreover, mass spectrometry analysis and Co-IP assays revealed an interaction between Cop1 and Rhbdf2. Furthermore, we found that overexpression of Cop1 can inhibit the protein expression levels of Rhbdf2 and p-TAK1. In fact, in vitro ubiquitination experiments revealed that Cop1 binds to Rhbdf2 and mediates its K48-linked ubiquitination and degradation. Together with the previous conclusions, these findings show that Cop1 overexpression leads to a decrease in Rhbdf2 protein level, which inhibits TAK1 phosphorylation and subsequently inhibits the JNK/p38 signaling pathway, ultimately exerting its protective effect. Our rescue experiments showed that overexpression of Rhbdf2 could significantly inhibit the protective effect of Cop1 overexpression on HIRI, which further confirmed that Cop1 inhibited the TAK1-JNK/p38 axis by mediating the ubiquitination and degradation of Rhbdf2, thereby alleviating HIRI.

In summary, our study revealed the molecular mechanism by which Rhbdf2 exerts its damaging effects in HIRI and suggests a new insight that Cop1-mediated Rhbdf2 ubiquitination and degradation inhibit the TAK1-JNK/p38 axis, thereby alleviating HIRI. This provides potential targets and new ideas for the treatment of HIRI. However, this study still has certain limitations. First, the binding site between Cop1 and Rhbdf2 needs to be determined. Second, whether Rhbdf2 regulates the phosphorylation of TAK1 in HIRI through direct binding remains to be elucidated. Future research will focus on exploring its detailed mechanisms. In summary, our study found that Cop1-regulated Rhbdf2 participates in HI/R-induced liver injury, inflammatory response, and apoptosis through the TAK1-JNK/p38 axis, providing potential targets and theoretical basis for the clinical treatment and relief of HIRI.

## Materials and Methods

### Human Liver Transplant Samples

This study used liver tissue specimens from 16 brain-dead donors. During the pre-transplant evaluation period, liver biopsy specimens were collected from the right lobe margin of the donor liver for use as the Pre group. Before closing the abdominal cavity, intraoperative biopsy specimens were collected from the right lobe margin of the transplanted liver for use as the Post group. All patients signed informed consent forms. This study adhered to the principles of the 1975 Declaration of Helsinki and the ethical guidelines of the Human Ethics Committee of the First Affiliated Hospital of Zhengzhou University (Zhengzhou, China) (2024-KY-0841-001). Patient information is detailed in Table 1. The GSE112713 and GSE12720 datasets were obtained from the GEO database (https://www.ncbi.nlm.nih.gov/geo) and used in this experimental study.

**Table 1:** Baseline Characteristics of Liver Transplantation Donors

No.	Age, *years*	Gender	Height, *cm*	Weight, *kg*	BMI, k*g/m*^*2*^	Cause of death	Previous condition	Regeneration time, *minutes*	Graft type	Sample tested in
1	54	Female	158	58	23.23345618	Head trauma	Not reported	370	Orthotopic liver transplant	qRT-PCR, Western blot
2	58	Female	156	55	22.60026298	Intracerebral hemorrhage	Not reported	376	Orthotopic liver transplant	qRT-PCR, Western blot
3	34	Female	158	40	16.02307323	Intracerebral hemorrhage	Not reported	321	Orthotopic liver transplant	qRT-PCR, Western blot
4	65	Female	163	55	20.70081674	Intracerebral hemorrhage	Not reported	400	Orthotopic liver transplant	qRT-PCR, Western blot
5	54	Male	172	70	23.66143862	Head trauma	Not reported	362	Orthotopic liver transplant	qRT-PCR, Western blot
6	19	Male	178	70	22.09317005	Head trauma	Not reported	410	Orthotopic liver transplant	qRT-PCR, Western blot
7	40	Male	175	75	24.48979592	Intracerebral hemorrhage	Not reported	383	Orthotopic liver transplant	qRT-PCR, Western blot
8	33	Male	173	70	23.38868656	Intracerebral hemorrhage	Not reported	317	Orthotopic liver transplant	qRT-PCR, Western blot
9	51	Male	174	78	25.76298058	Stroke	Not reported	365	Orthotopic liver transplant	qRT-PCR, Western blot
10	55	Male	175	70	22.85714286	Stroke	Not reported	336	Orthotopic liver transplant	qRT-PCR, Western blot
11	34	Male	178	90	28.40550436	Head trauma	Not reported	396	Orthotopic liver transplant	qRT-PCR, Western blot
12	60	Male	172	65	21.97133586	Head trauma	Not reported	565	Orthotopic liver transplant	qRT-PCR, Western blot
13	20	Male	170	65	22.49134948	Glioma	Not reported	453	Orthotopic liver transplant	qRT-PCR, Western blot
14	34	Male	178	80	25.2493372	Head trauma	Not reported	192	Orthotopic liver transplant	qRT-PCR, Western blot
15	38	Male	170	85	29.41176471	Aortic dissection	Not reported	374	Orthotopic liver transplant	qRT-PCR, Western blot
16	49	Male	168	70	24.8015873	Intracerebral hemorrhage	Not reported	277	Orthotopic liver transplant	qRT-PCR, Western blot

BMI, body mass index; qRT-PCR, quantitative real-time polymerase chain reaction.

### Animals

The male C57BL/6J mice (8 ± 1 weeks old; 22 ± 2 g) used in this study were purchased from Vital River, and were raised in a specific pathogen-free (SPF) facility with a 12-hour/12-hour light/dark cycle, and ad libitum access to sterilized food and water. All mice were acclimated to the environment for 1 week before the experiment began. All mice experiments in this study were approved by the Ethics Committee of the First Affiliated Hospital of Zhengzhou University (2024-KY-0841-001).

### HI/R Mouse Model

We used a portion (70%) of the warm HI/R model.^30^^,^^41^^,^^42^ Mice were anesthetized by intraperitoneal injection of 1% sodium pentobarbital at a dose of 50 mg/kg after 12 hours of fasting. After immobilizing the mouse, the abdominal cavity was opened along the midline to expose the liver, and the liver vessels were meticulously dissected under a microscope. Microvascular clamps were used to occlude the vessels supplying the middle and left sides of the liver of the mice in the HI/R group. After 60 minutes of occlusion, the microvascular clamps were removed, and blood flow was restored for 3, 6, and 12 hours. Blood samples and liver samples were collected for subsequent assays. For the sham group, the abdominal cavity was opened, and the liver vessels were only isolated without any other treatment.

### Detection of Liver Damage

Serum was collected from the blood of sham group and HI/R group mice after separation by centrifugation. The levels of ALT and AST in the serum were measured using Aspartate Aminotransferase and Alanine Aminotransferase Assay Kits (Jiancheng).

### H&E Staining

Fixed mouse liver tissue specimens were fixed with tissue fixative solution (10% formalin), and then dehydrated, embedded in paraffin, and sectioned into 5-μm thick continuous paraffin sections. After staining the sections with H&E using an H&E staining kit (Servicebio), following the instructions of the manufacturer of the kit. Ultimately, random images were captured using an Olympus optical microscope (Olympus Corporation).

### TUNEL Assay

After cutting the paraffin sections (as described above), the paraffin-embedded sections were deparaffinizes with xylene for 5 minutes and washed with anhydrous ethanol. Then, after adding DNase-free proteinase K (Beyotime), the sections were incubated at 37°C for 15 minutes. Subsequently, after washing 3 times with phosphate-buffered saline (PBS), the TUNEL assay detection solution (Beyotime) was added, and sections were incubated in the dark for 1 hour. Then, after washing 3 times with PBS, cell nuclei were counterstained with 4′,6-diamidino-2-phenylindole (DAPI; Beyotime). Finally, the sections were observed under a fluorescence microscope, and images were captured randomly.

### Immunofluorescence Staining

Paraffin sections were dewaxed, rehydrated, and processed for antigen retrieval using an ethylenediaminetetraacetic acid (EDTA) antigen retrieval solution (Servicebio), according to the manufacturer’s protocol. Then, the sections were incubated in 10% fetal bovine serum (FBS) at room temperature for 60 minutes, followed by washing 3 times with PBS. Subsequently, the sections were incubated with anti-mouse Ly6G and CD11b (1:100; Servicebio) at 4°C for 12 hours, and then washed 3 times with PBS, followed by incubation with the appropriate fluorescent secondary antibody at 37°C for 60 minutes. After washing 3 times with PBS, the cell nuclei were stained with DAPI, and the cells were eventually observed under a fluorescence microscope and images of randomly selected fields of view were captured.

### Cell Culture and H/R

The normal mouse liver cell line AML12 was purchased from Cellcook Biotech and was checked for mycoplasma contamination before use. The AML12 cells were cultured in Dulbecco’s Modified Eagle’s Medium (DMEM)/F12 medium supplemented with 1 × ITS (insulin, transferrin, and selenium), 40 ng/mL dexamethasone, and 10% FBS in a humidified incubator at 37°C with 5% CO_2_. Once the cells had adhered and reached 80% (±5%) confluence, the medium was replaced with fresh DMEM/F12 medium without glucose or serum. The cells were then subjected to hypoxia for 6 hours in a hypoxic workstation at 37°C, 1% O_2_, 5% CO_2_, and 94% N_2_. After reoxygenation for 3, 6, and 12 hours, the cells were collected for subsequent assays.

### Construction of AAV and Plasmids

Rhbdf2 overexpression (HA-Rhbdf2) and knockdown plasmids were purchased from the Public Protein/Plasmid Library. The Cop1 overexpression plasmid (Flag-Cop1) was purchased from the MiaoLing Plasmid Platform. The target plasmid and packaging plasmids (Pmd2.G and psPAX2) were co-transfected into HEK293T cells using Lipo8000 transfection reagent (Beyotime). After 48 hours of transfection, the HEK293T cell supernatant was collected to obtain the virus. The collected virus particles were transfected into AML12 cells for 24 h. Then, the medium was replaced with fresh medium containing 2 μg/mL puromycin (Beyotime), and selection with puromycin was performed for 5 days to establish stably transfected cell lines. Ultimately, cell lines stably transfected with specific plasmids were identified by qRT-PCR and Western blot analysis. Rhbdf2 overexpression (AAV-Rhbdf2) and Rhbdf2 knockdown (AAV-shRhbdf2) AAVs were purchased from Obio Technology Co, Ltd. Rhbdf2 knockdown and overexpressing mice were constructed by tail vein injection according to the instructions provided by the Manufacturer (Obio Technology Co, Ltd), and the successful construction of transgenic mice was verified after 4 weeks.

### Co-IP and Mass Spectrometry Assays

For Co-IP assays, AML12 and HEK293T cells were first co-transfected with the designated plasmid for 1 day. After co-transfection, cells were collected and lysed using cell IP Lysis buffer to lyse the cells. After sonication, the cell lysate was centrifuged, and the supernatant was collected. Then, the supernatant was incubated with protein A/G agarose beads (Beyotime) and the corresponding fluorescently labeled antibody at 4°C for 12 hours. Subsequently, after washing the magnetic beads 3 times with NaCl buffer, sodium dodecyl sulfate-polyacrylamide gel electrophoresis (SDS-PAGE) loading buffer (Solarbio) was added, and the mixture was boiled at 95°C for 15 minutes. After separating the proteins by SDS-PAGE, the proteins were transferred to a polyvinylidene fluoride (PVDF) membrane, and the immunoreactive proteins were detected by performing Western blot analysis. For mass spectrometry analysis, Rhbdf2 overexpressing stable cell lines were subjected to H/R, protein samples were collected as described above, proteins were separated by SDS-PAGE on a 10% acrylamide gel, and subsequently gels with the separated proteins were stained with silver staining using the Pierce Silver Staining kit (Pierce/Thermo Fisher Scientific Inc). Finally, the stained separated proteins on the gel were further analyzed by liquid chromatography-tandem mass spectrometry (LC-MS/MS).

### Flow Cytometry Analysis

After being subjected to H/R, cells were digested with EDTA-free 0.25% trypsin (Beyotime), collected, washed 3 times with PBS, and resuspended in 200 μL of binding buffer. Then, cells were fluorescently stained with 7-aminoactinomycin D (7-AAD) and phycoerythrin (PE) for 30 minutes, following the protocol of the apoptosis detection kit (Beyotime). The viability of the cells was then analyzed by flow cytometry.

### RNA-Seq Analysis

After subjecting Rhbdf2 negative controls and knockdown cell lines to H/R, total RNA was extracted and used to construct cDNA libraries, and quality control of the cDNA libraries was performed. Sequencing of the libraries was performed on the Illumina NovaSeq platform (Illumina Inc), according to the manufacturer’s instructions. Differential gene expression was determined using DESeq2, and genes with corrected *P*-values < .01 and fold changes ≥2 were designated as DEGs. GO term enrichment analysis of DEGs was performed using the clusterProfiler package based on the Wallenius non-central hypergeometric distribution. Subsequently, KEGG pathway enrichment analysis of DEGs was performed using the KOBAS database and clusterProfiler software.

### qRT-PCR Analysis

After extracting total RNA from mouse liver tissue samples and AML12 cells using TRIzol reagent (Solarbio), the extracted total RNA was reverse transcribed into cDNA using the Vazyme reverse transcription kit (Vazyme) following the manufacturer’s instructions. Then, the cDNA was amplified using the Universal SYBR qPCR Master Mix (Biosharp), and the mRNA expression levels of the relevant genes were determined using the 2^-△△Ct^ method, using β-actin as the internal reference.^43^ The sequences of the relevant primers are listed in Table 2.

**Table 2:** Primers Used in This Study

Primer	Forward	Reverse
Rhbdf2 (mouse)	AACCCAGCCTACCTGAAGAGT	CGATGCCAGTTTTGTCGCTT
TNF-α	CCAAAGGGATGAGAAGTTCC	CTCCACTTGGTGGTTTGCTA
IL-6	CACATGTTCTCTGGGAAATCGTGGA	TCTCTCTGAAGGACTCTGGCTTTGT
IL-1β	CAAACGGGAAGGGATATGGG	CAGTGTGTGGGTTGCCTTAT
Rhbdf2 (human)	GATGGGGCAGACACGTTTGA	CCTCGGAAGTAGCTGGCAG
β-actin (mouse)	GTGACGTTGACATCCGTAAAGA	GCCGGACTCATCGTACTCC
β-actin (human)	ACCTTCTACAATGAGCTGCG	CCTGGATAGCAACGTACATGG

### Western Blot Analysis

Proteins from mouse liver tissue and AML12 cells were extracted using radioimmunoprecipitation assay (RIPA) lysis buffer (Solarbio). After adding a protease inhibitor mixture (Beyotime) to the cell lysate, the protein concentration was determined using a bicinchoninic acid (BCA) assay kit (Beyotime). Then, the proteins were separated by SDS-PAGE and transferred to a PVDF membrane. Subsequently, after blocking with 5% nonfat milk for 1 hour, the membrane was incubated with the corresponding primary antibody at 4°C for 12 hours, and after a brief wash, the membrane was then incubated with the appropriate secondary antibody at room temperature for 1 hour. The immunoreactive protein bands were visualized by detecting the protein chemiluminescence signals using an enhanced chemiluminescence (ECL) reagent (NCM Biotech Co, Ltd) and the ImageQuant system (Cytiva). Information about the antibodies used in this assay is listed in Table 3.

**Table 3:** Antibodies Used in This Study

Antibodies	Source	Identifier
Rhbdf2	Immunoway	YN6878
β-actin	Proteintech	66009-1-Ig
Cop1	Abcam	ab70889
TAK1	Huabio	ET1705-14
p-TAK1	Huabio	HA723662
IKKβ	Abways	CY6607
p-IKKβ	CST	2078T
IKBα	Proteintech	10268-1-AP
p65	Abmart	T55034S
p-p65	CST	3033T
BAX	Proteintech	50599-2-Ig
BCL2	Immunoway	YM8319
C-caspase3	CST	9661T
p38	CST	9212S
p-p38	CST	4511T
JNK	CST	9252T
p-JNK	CST	4668T
ERK	CST	4695T
p-ERK	CST	4370T
HA	MBL	M180-3
Flag	MBL	M185
Myc	Vazyme	RA1005-01

### Statistical Analysis

Statistical analysis was performed using the GraphPad Prism 8.0 software (GraphPad Software Inc), and the experimental data are presented as the mean ± standard error of the mean (SEM). Before data analysis, the Shapiro-Wilk test was performed to determine whether the data were normally distributed, followed by Levene’s test to assess homogeneity of variance. If the data were normally distributed and had equal variances, an independent samples *t*-test was used; if the data were normally distributed but had unequal variances, a Welch *t*-test was used; if the data were not normally distributed, a Mann-Whitney *U* test was used. The significance level was set at *P* < .05.

## Supplementary Material

Supplementary Material

Extended PDF
