# Supplementary material for: Ubiquitination of Rhomboid 5 Homolog 2 by Constitutive Photomorphogenic 1 Alleviates Hepatic Ischemia-reperfusion Injury by Regulating the Transforming Growth Factor-β Activating Kinase 1-C-Jun N-terminal Kinase/p38 Signaling Pathway
Source: Cell Mol Gastroenterol Hepatol. 2025 Dec 5;20(4):101695. doi: 10.1016/j.jcmgh.2025.101695 (PMC12873737; doi:10.1016/j.jcmgh.2025.101695)

Figure 1

Figure 1A

Human 1-4 Rhbdf2

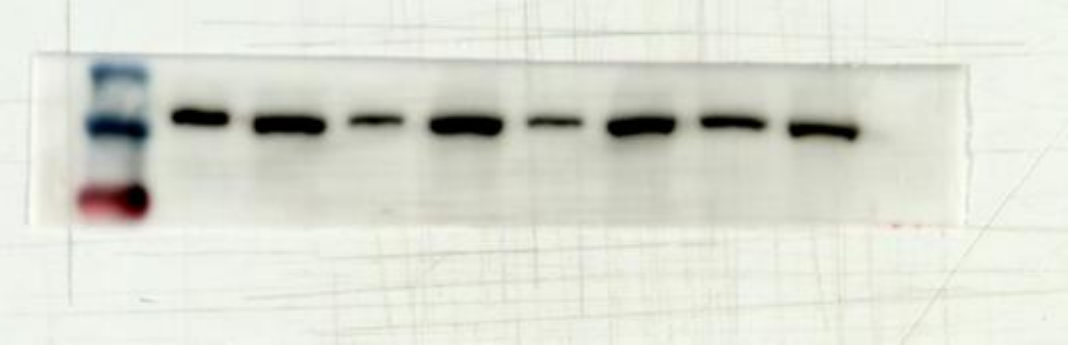

Human 1-4 β-actin

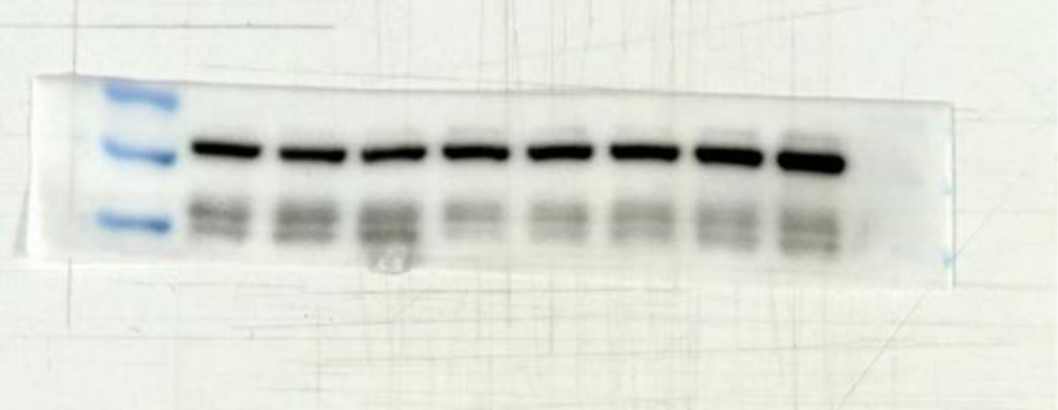

Human 5-8 Rhbdf2

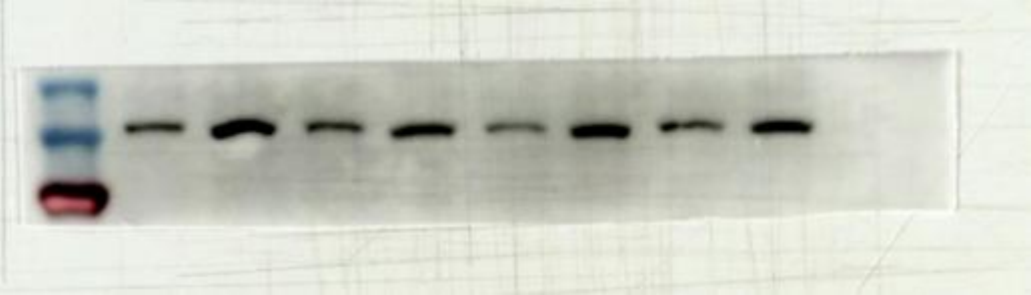

Human 5-8 β-actin

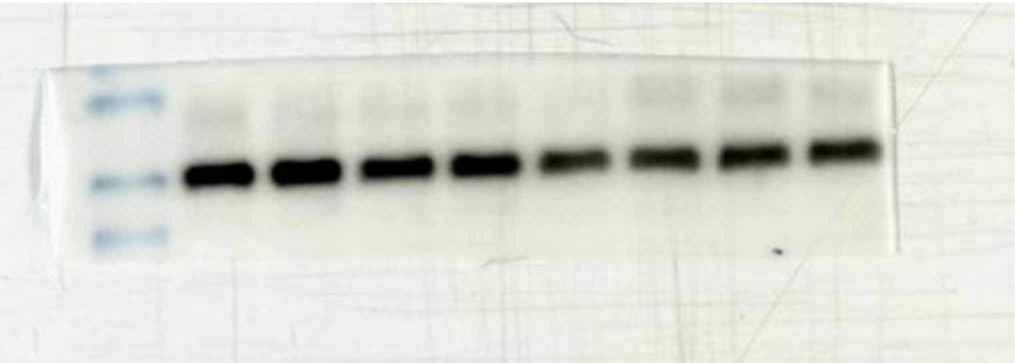

Human9-12 Rhbdf2

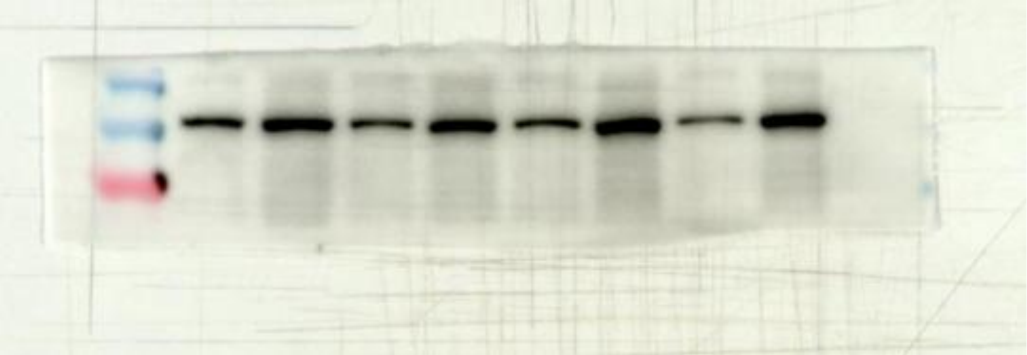

Human 9-12 β-actin

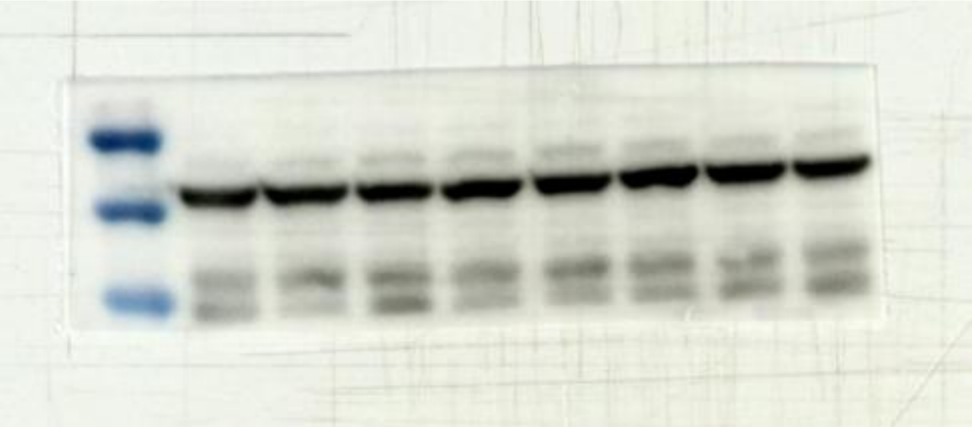

Human 13-16 Rhbdf2

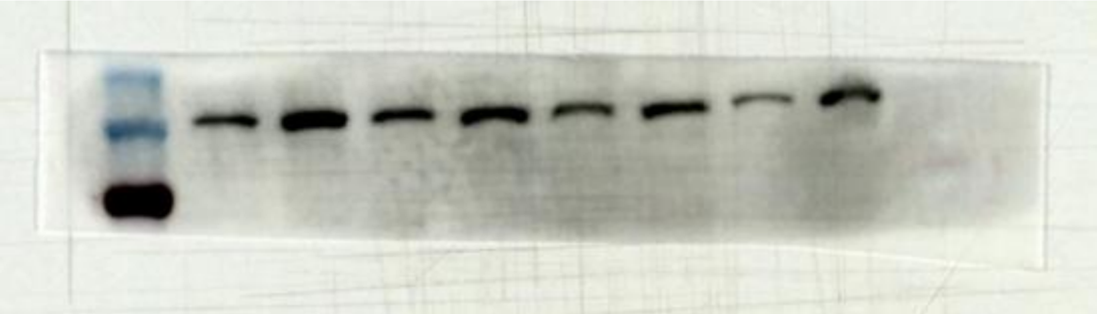

Human 13-16 β-actin

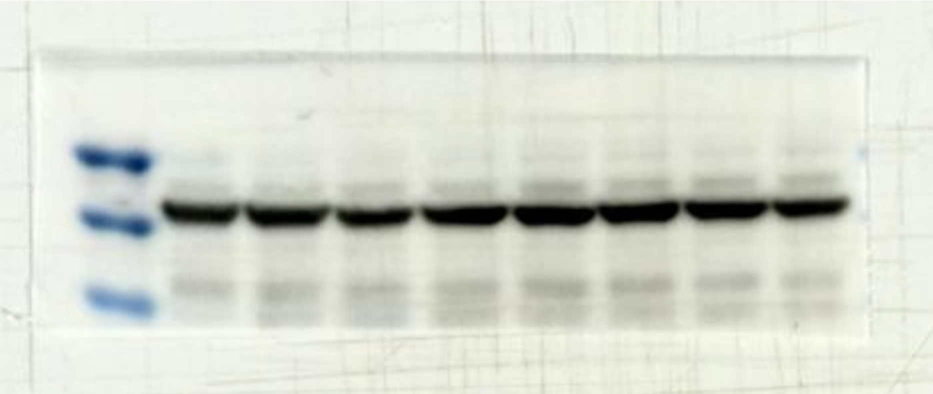

Figure 1D

Rhbdf2

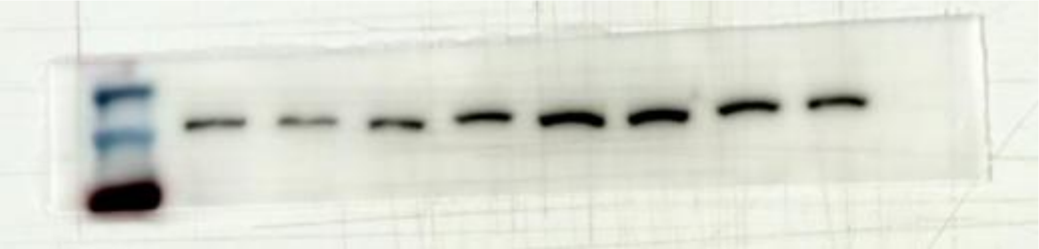

β-actin

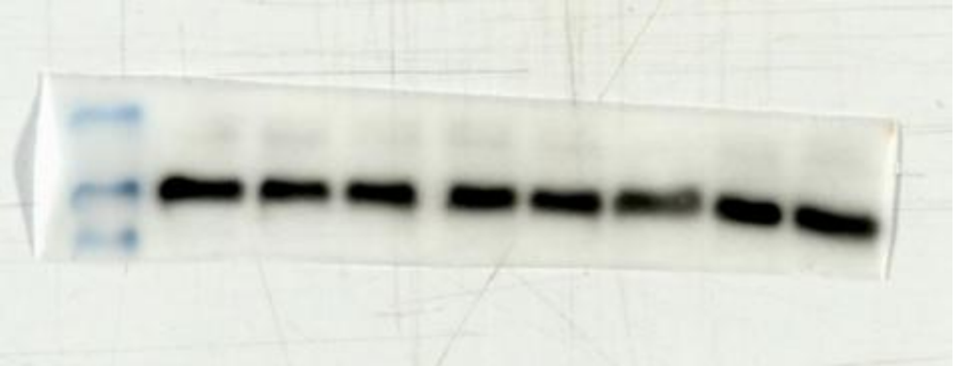

Figure 1E

Rhbdf2

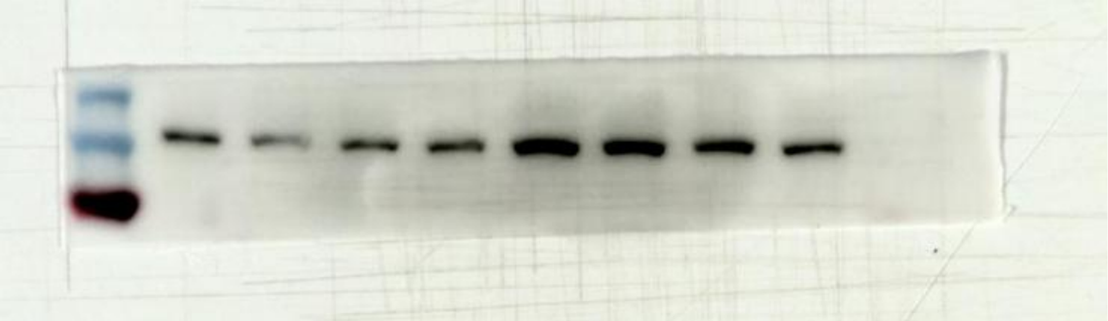

β-actin

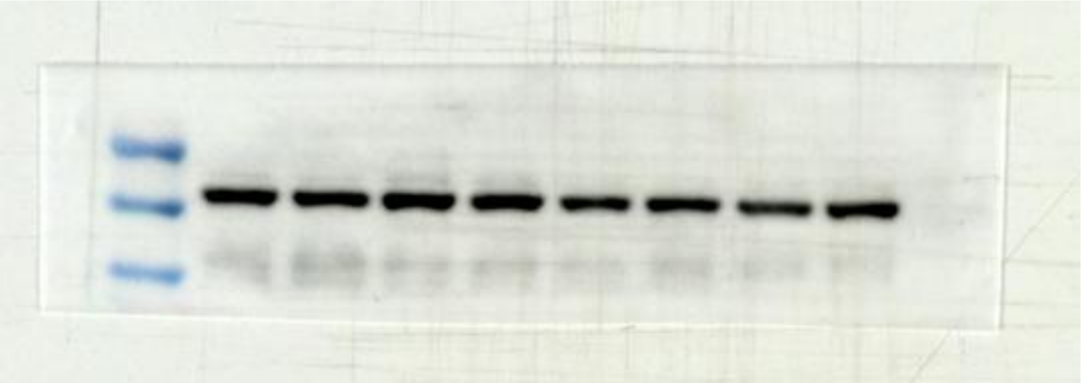

Figure 2

Figure 2A
Rhbdf2

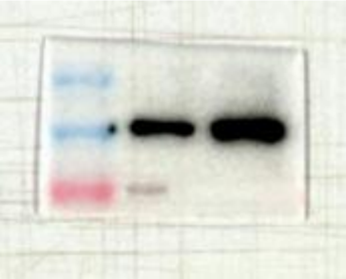

β-actin

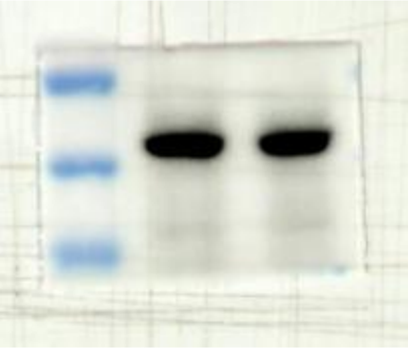

Figure 2B
Bax

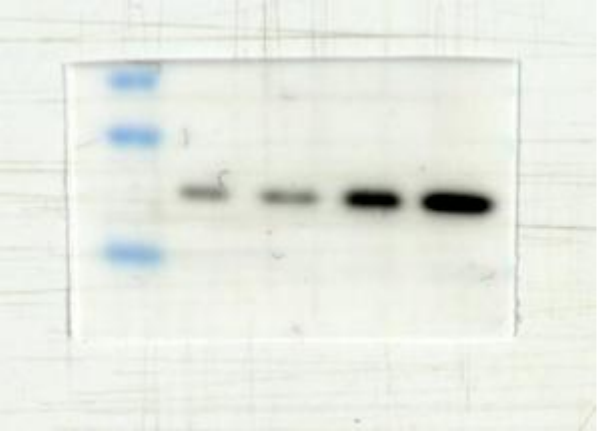

Bcl2

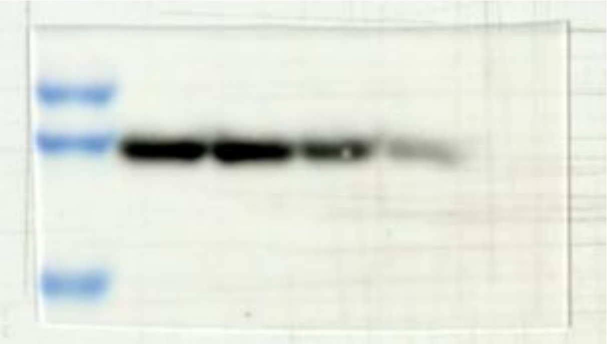

C-caspase3

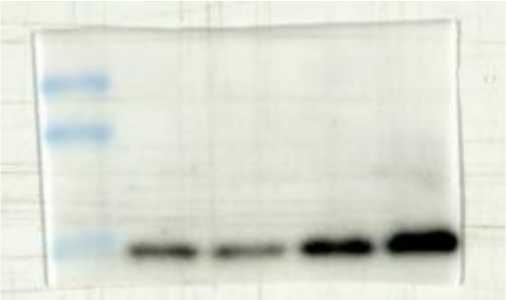

β-actin

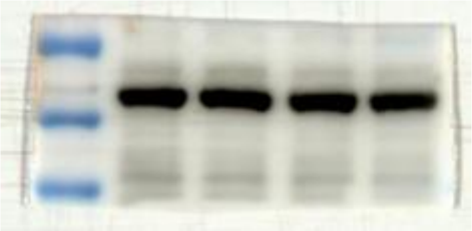

Figure 2E

IKKβ

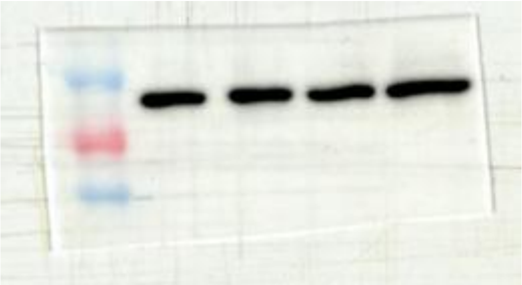

p-IKKβ

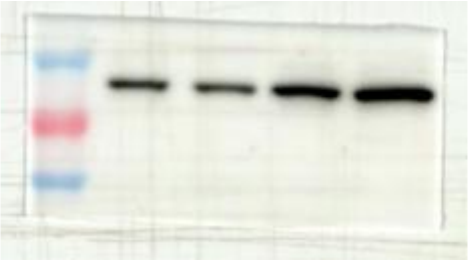

IKBα

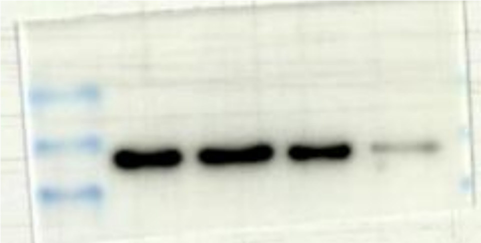

P65

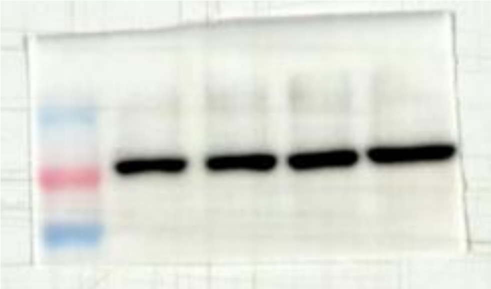

p-p65

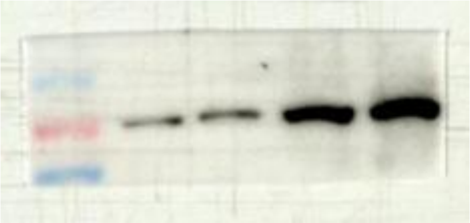

β-actin

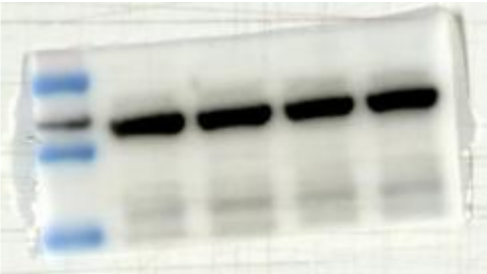

Figure 2F
Rhbdf2

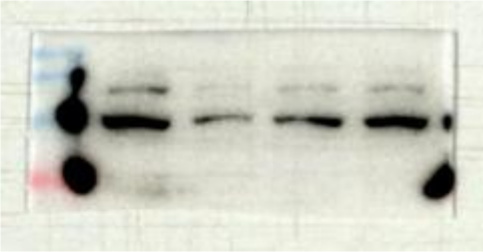

β-actin

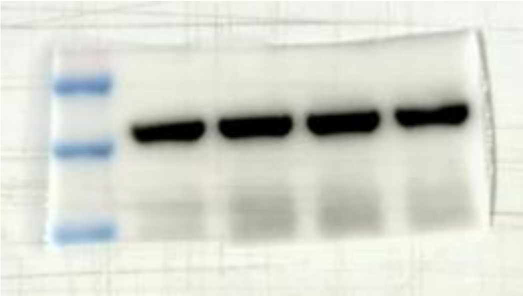

Figure 2G

Bax

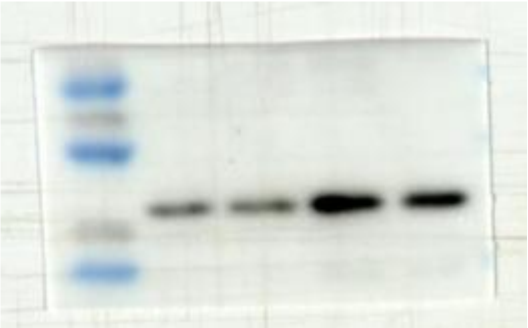

Bcl2

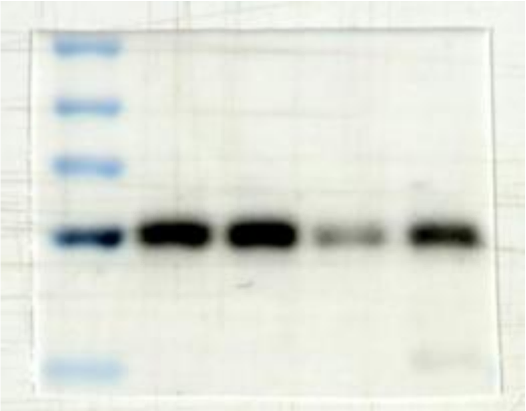

C-caspase3

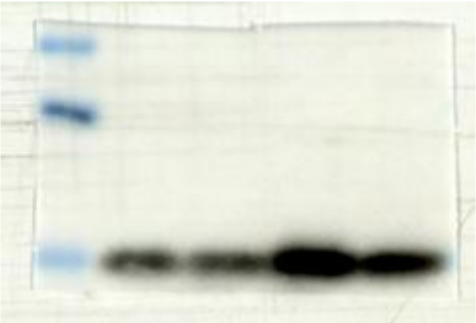

β-actin

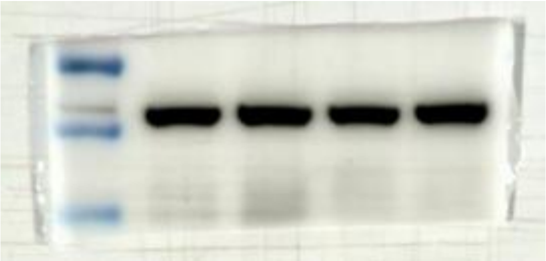

Figure 2J

IKKβ

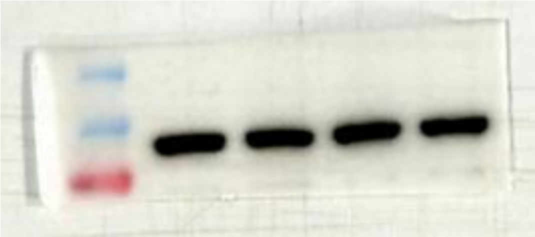

p-IKKβ

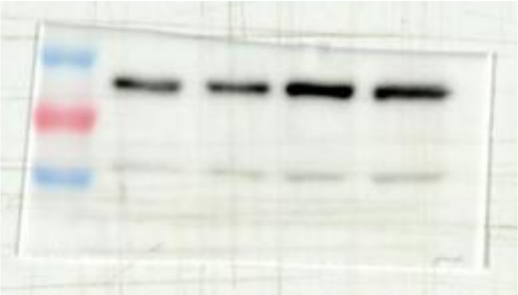

IKBα

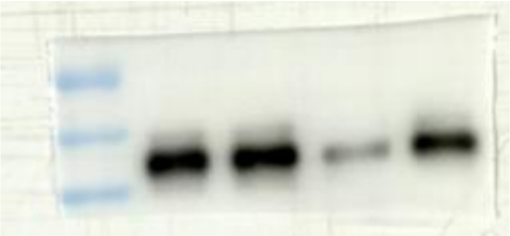

p-65

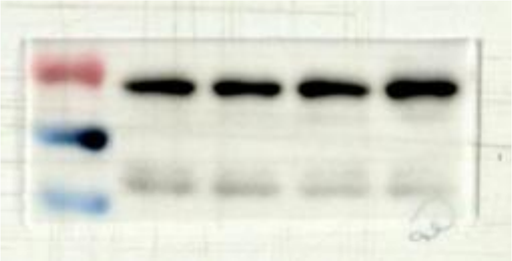

p-p65

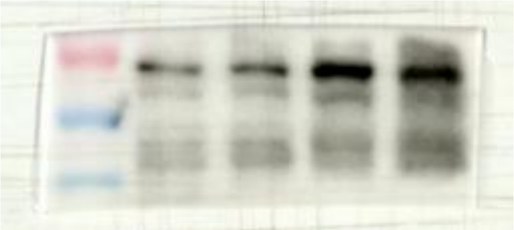

β-actin

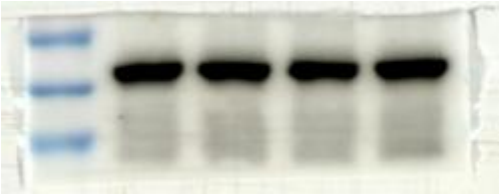

Figure 3

Figure 3A

Rhbdf2

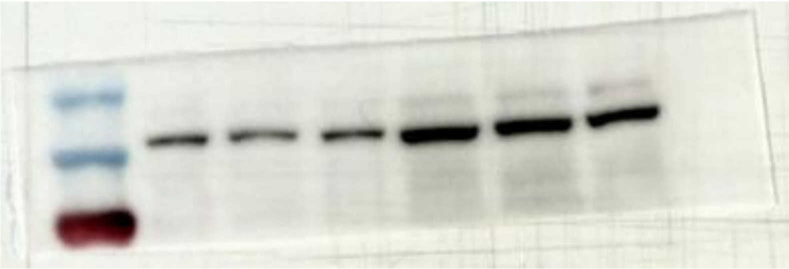

β-actin

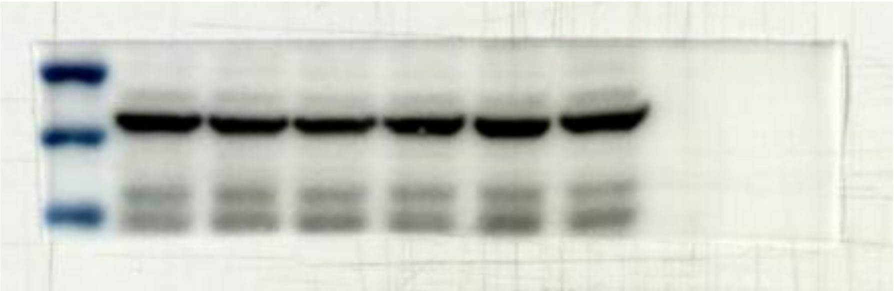

Figure 3F

Bax

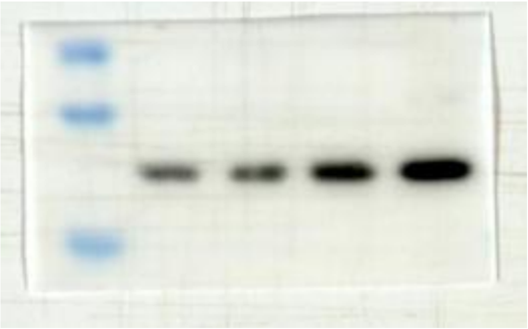

Bcl2

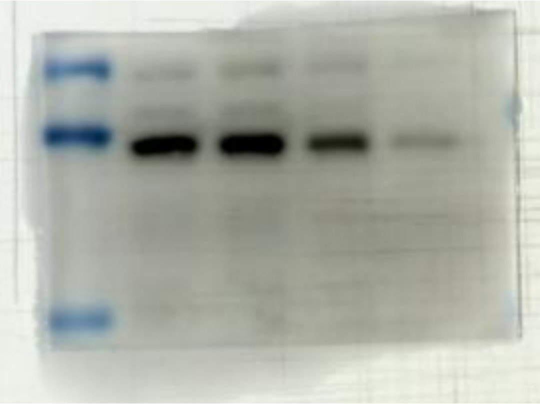

C-caspase3

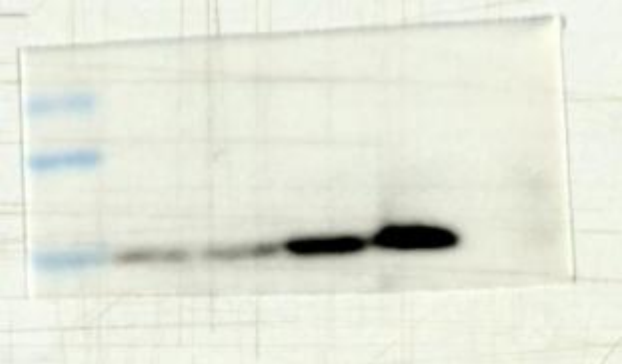

β-actin

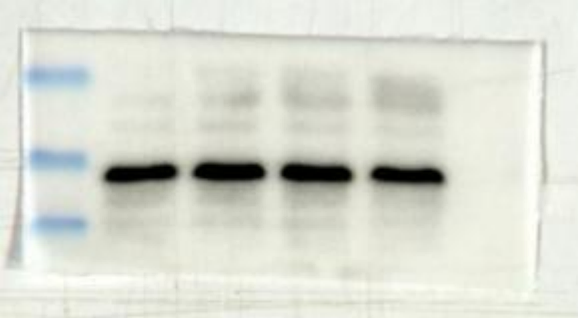

Figure 3J

IKKβ

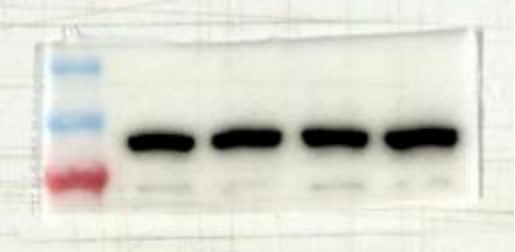

p-IKKβ

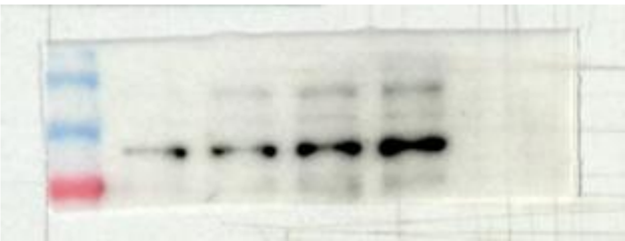

IKBα

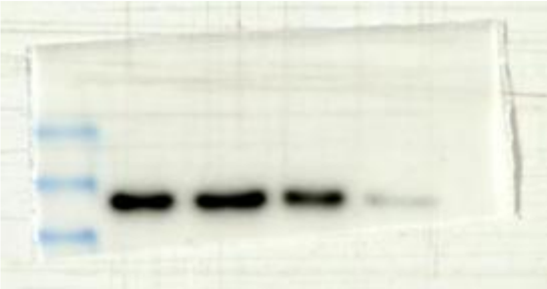

P65

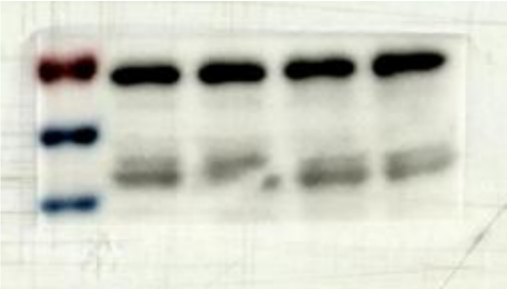

p-p65

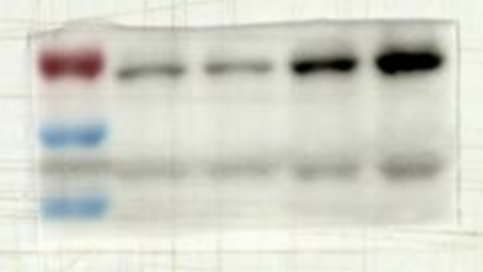

β-actin

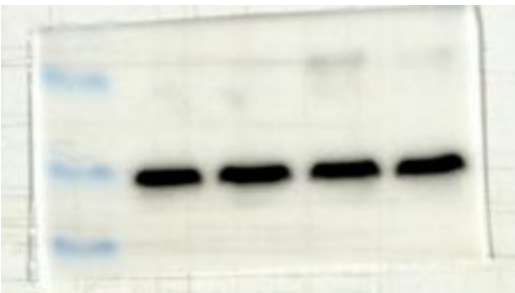

Figure 4

Figure 4A

Rhbdf2

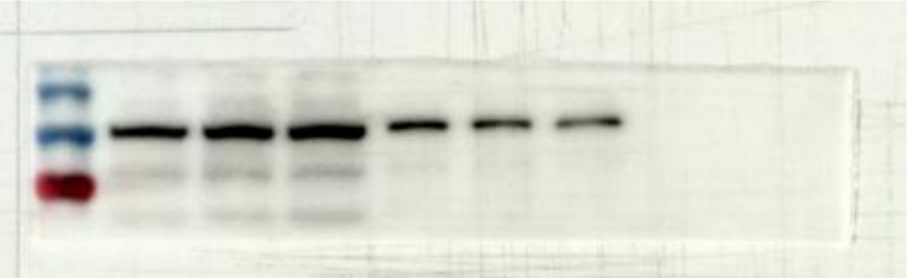

β-actin

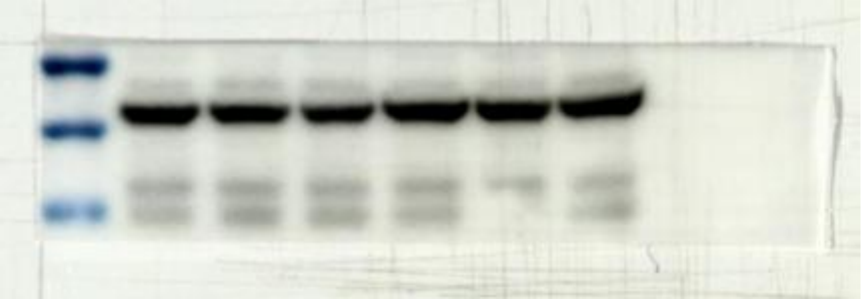

Figure 4F

Bax

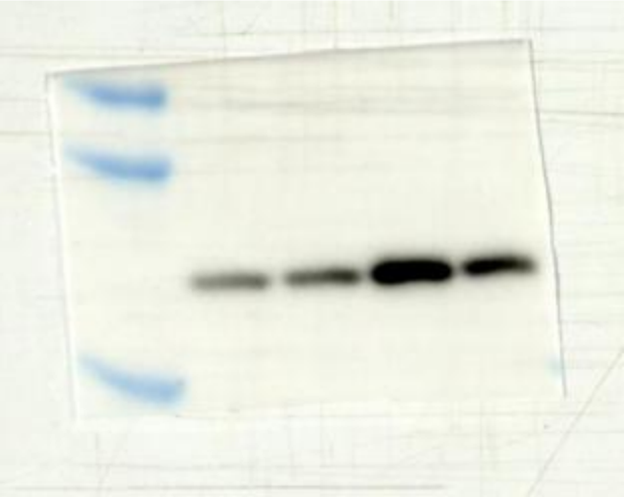

Bcl2

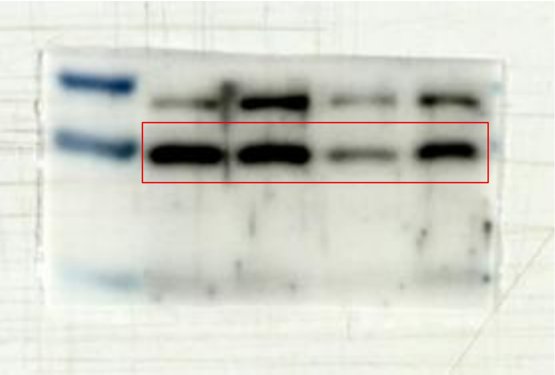

C-caspase3

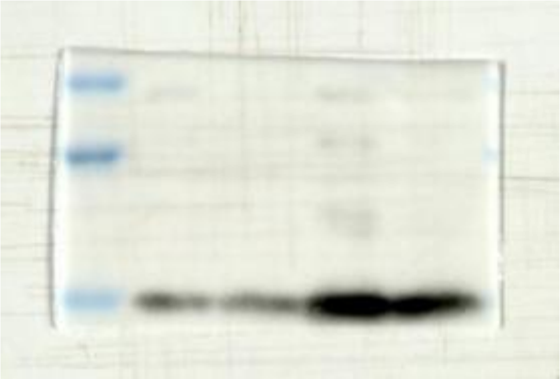

β-actin

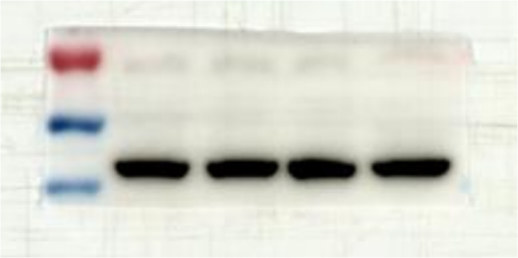

Figure 4J

IKKβ

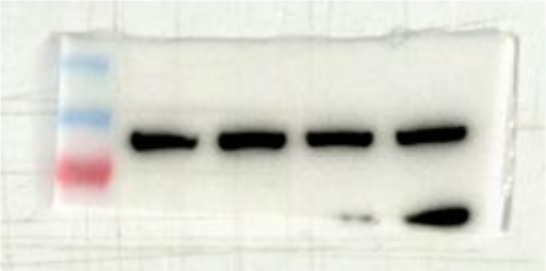

p-IKKβ

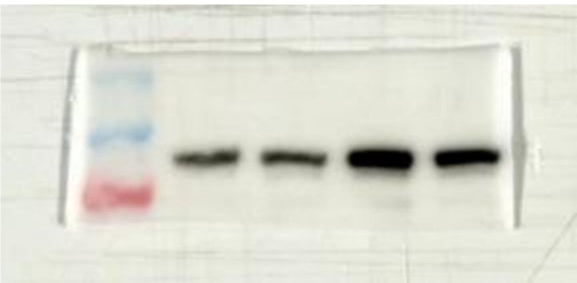

IKBα

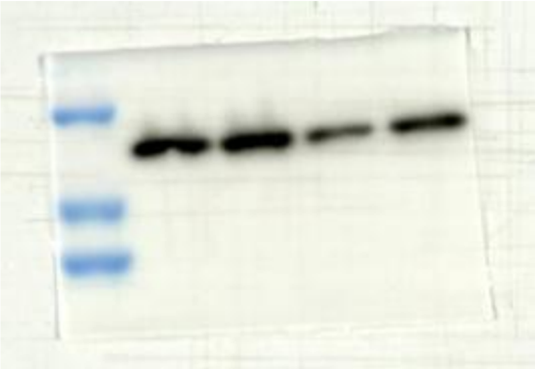

p65

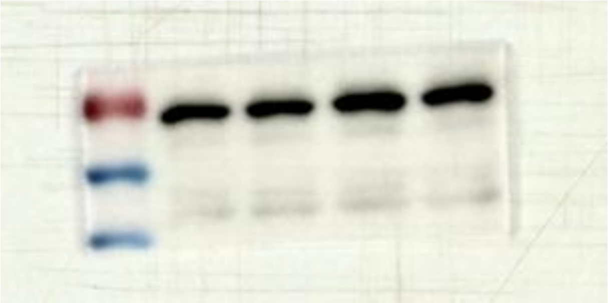

p-p65

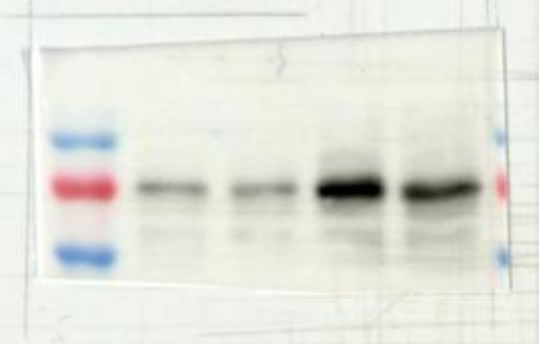

β-actin

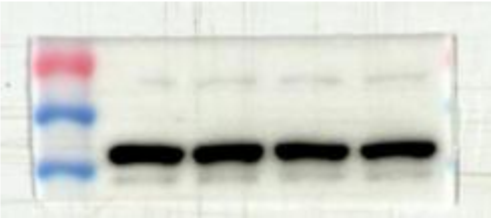

Figure 5

Figure 5A
Rhbdf2

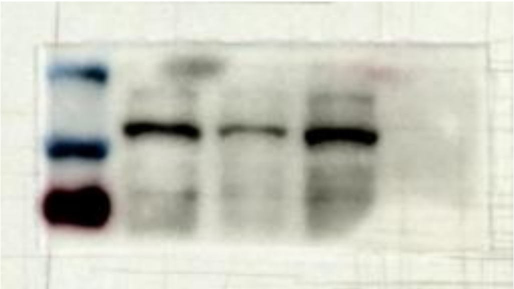

β-actin

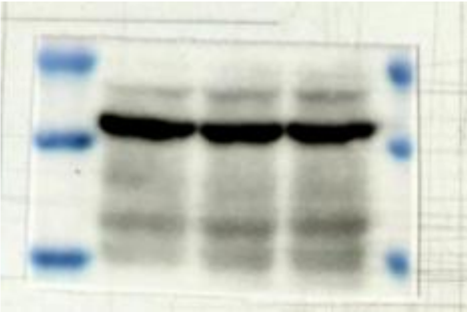

Figure 5C
Bax

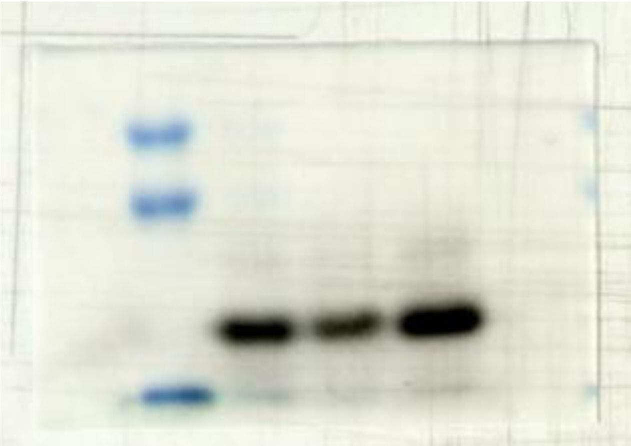

Bcl2

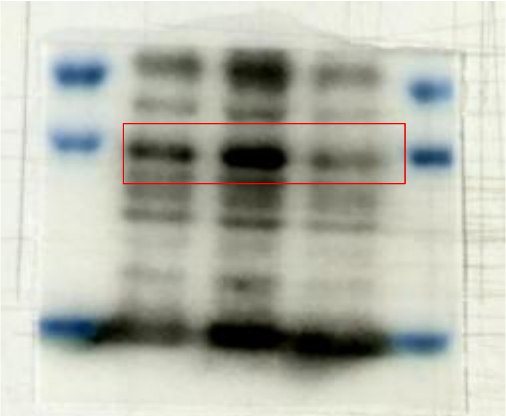

β-actin

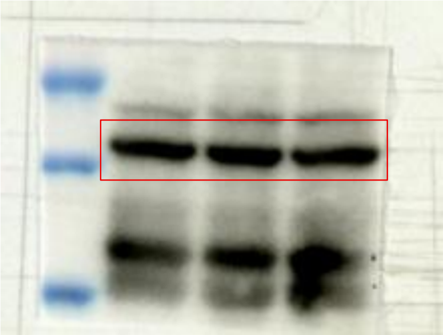

Figure 5D

IKKβ

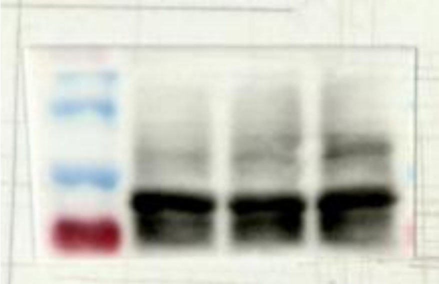

p-IKKβ

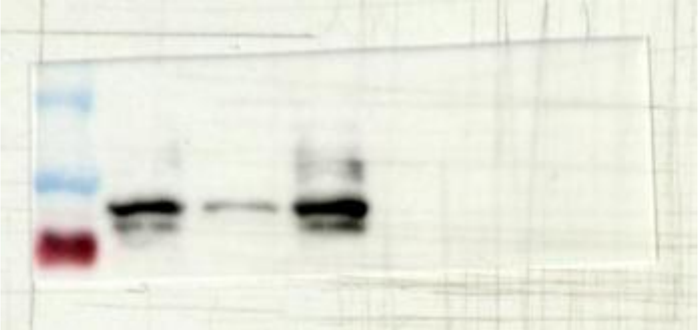

IKBα

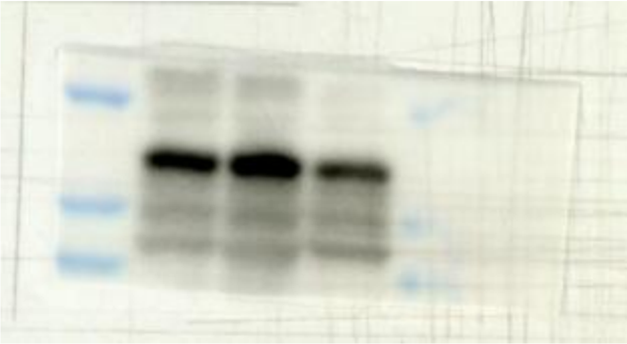

p65

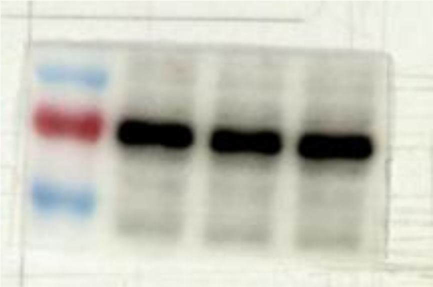

p-p65

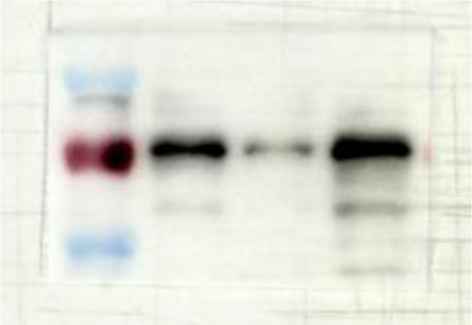

β-actin

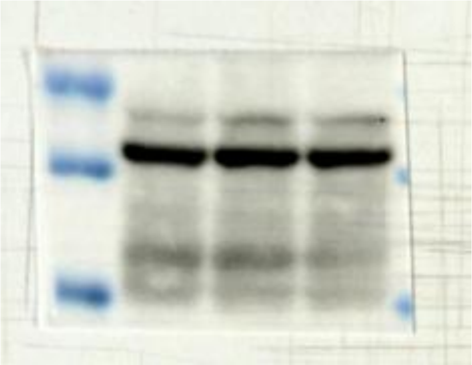

Figure 6

Figure 6D

TAK1

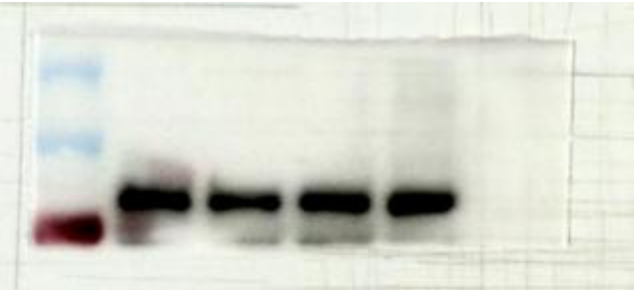

p-TAK1

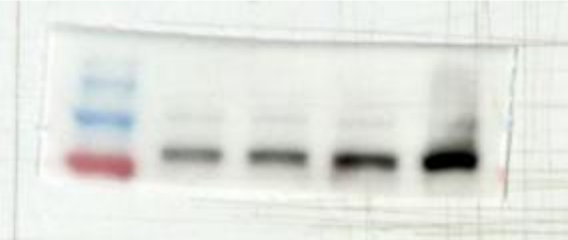

JNK

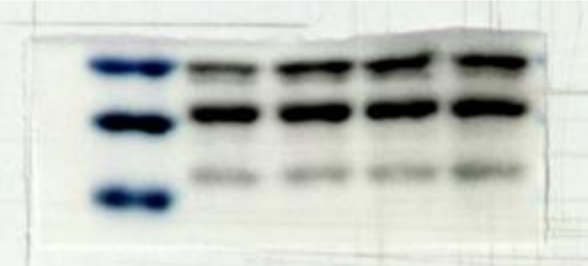

p-JNK

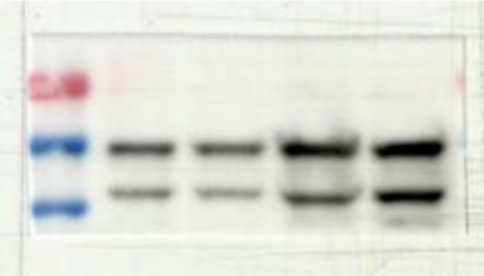

p38

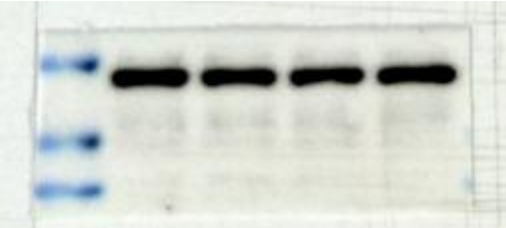

p-p38

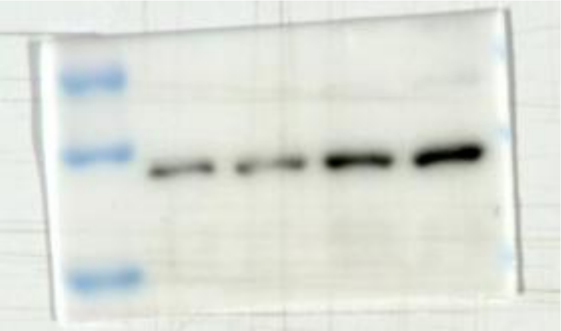

β-actin

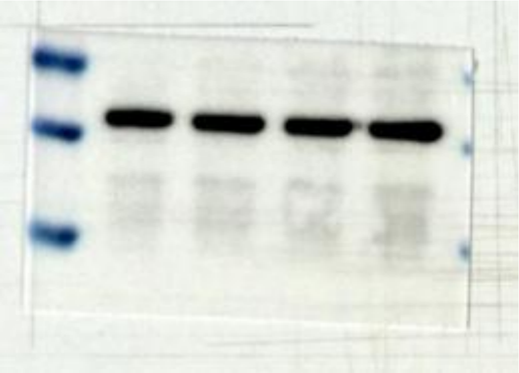

Figure 6E

TAK1

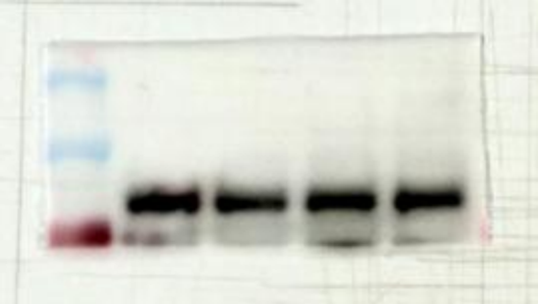

p-TAK1

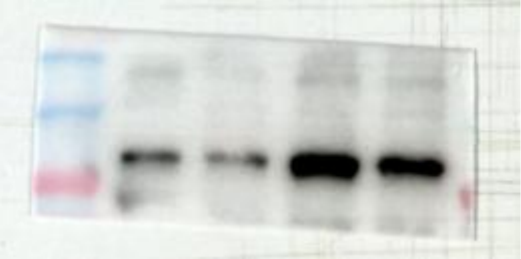

JNK

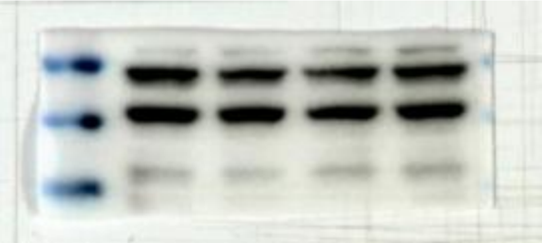

p-JNK

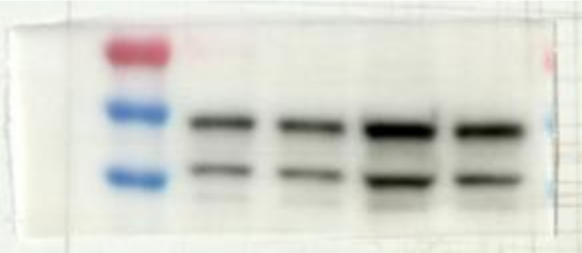

p38

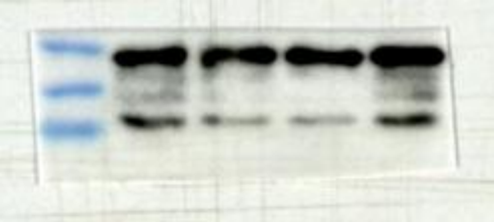

p-p38

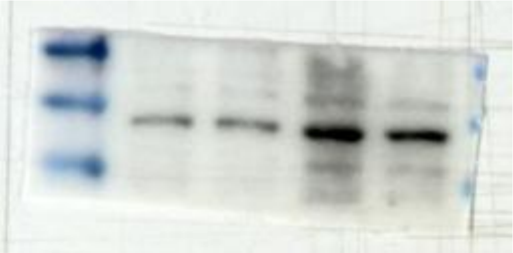

β-actin

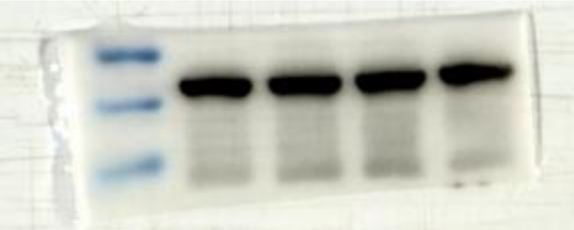

Figure 6F

OE ERK

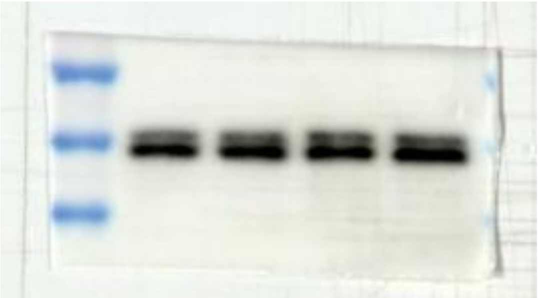

OE p-ERK

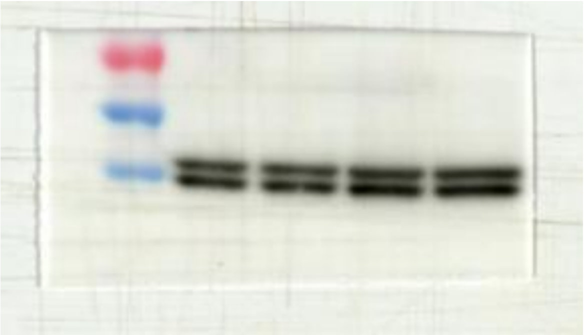

OE β-actin

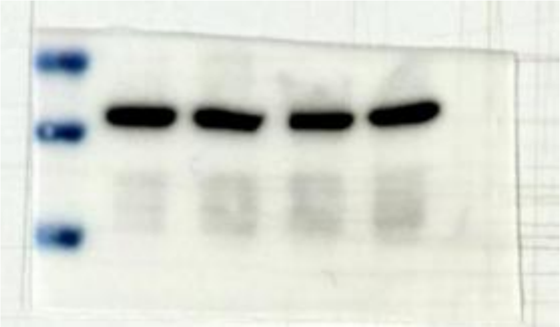

SH1 ERK

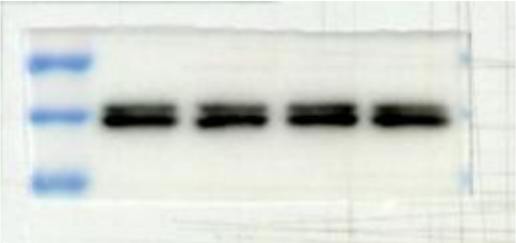

SH1 p-ERK

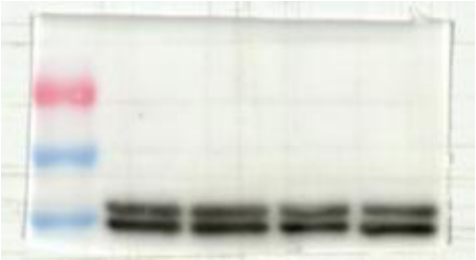

β-actin

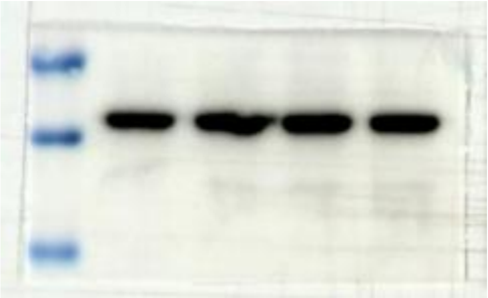

Figure 6G

TAK1

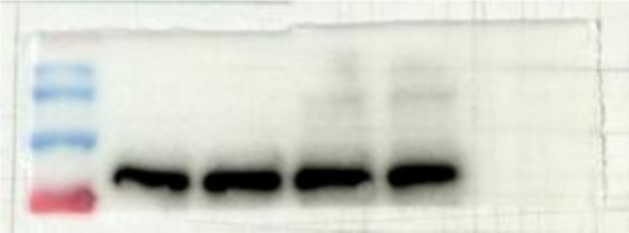

p-TAK1

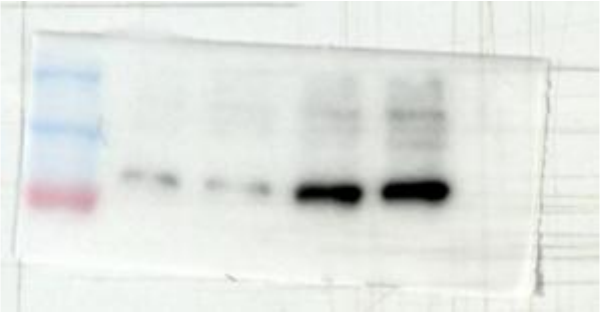

JNK

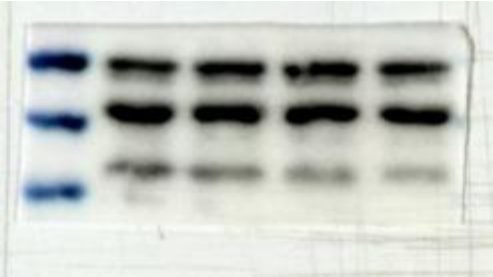

p-JNK

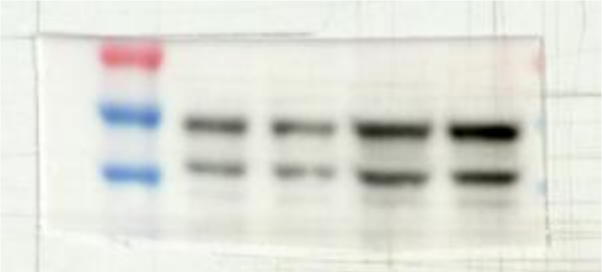

p38

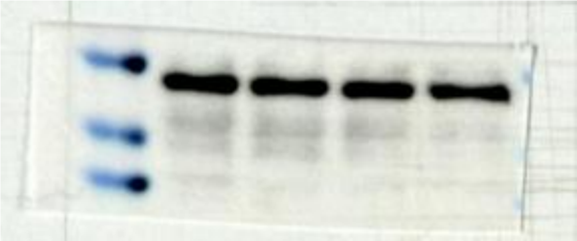

p-p38

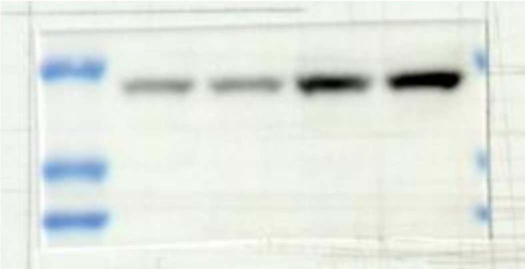

β-actin

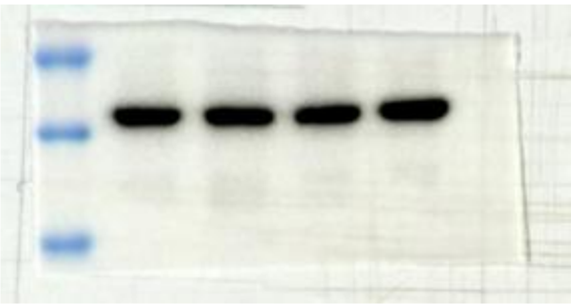

Figure 6H
TAK1

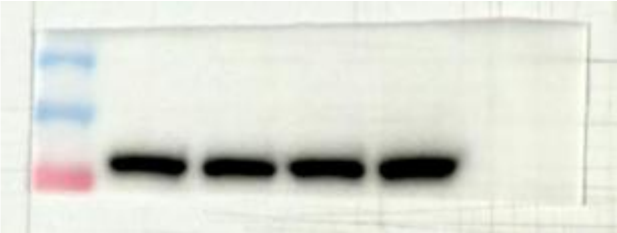

p-TAK1

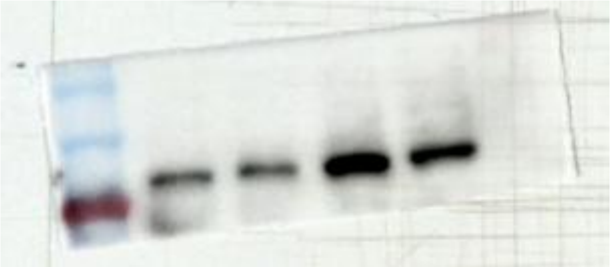

JNK

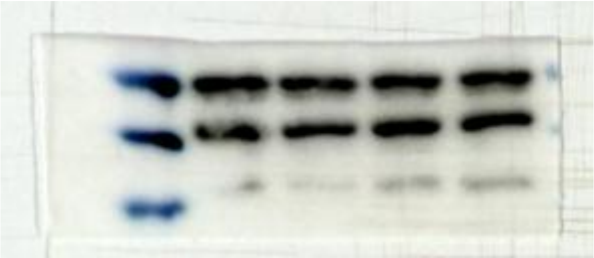

p-JNK

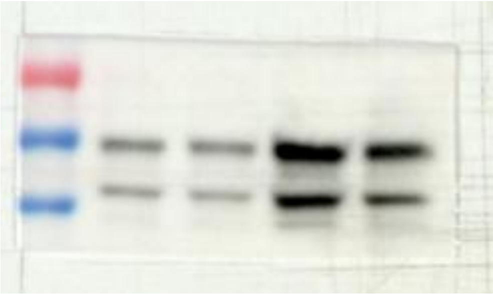

p38

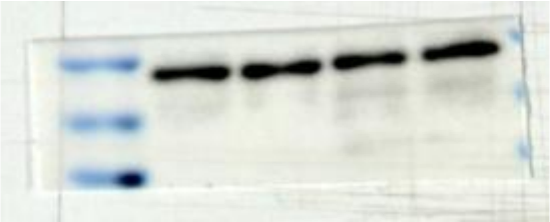

p-p38

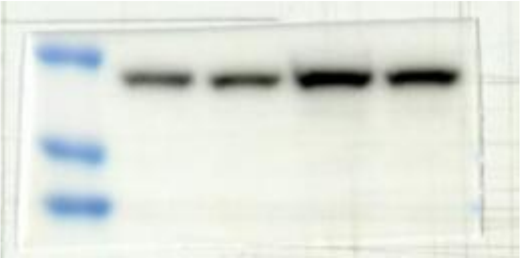

β-actin

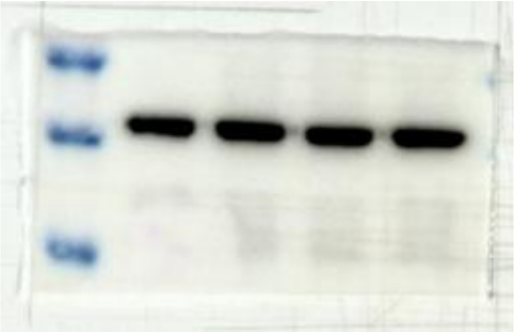

Figure 7

Figure 7A

Rhbdf2

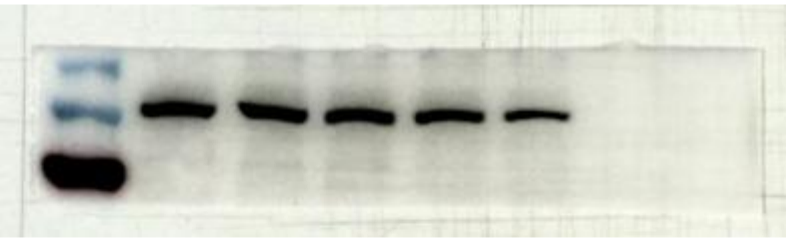

β-actin

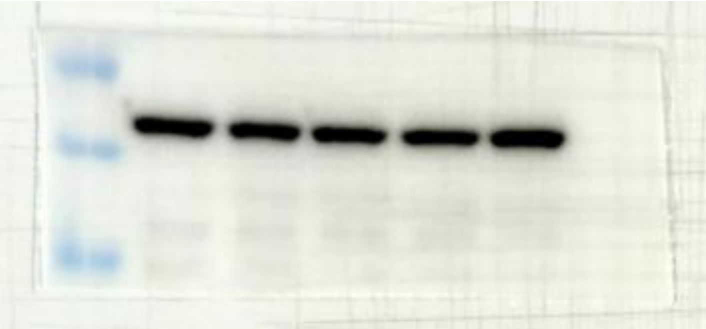

Figure 7B

Rhbdf2

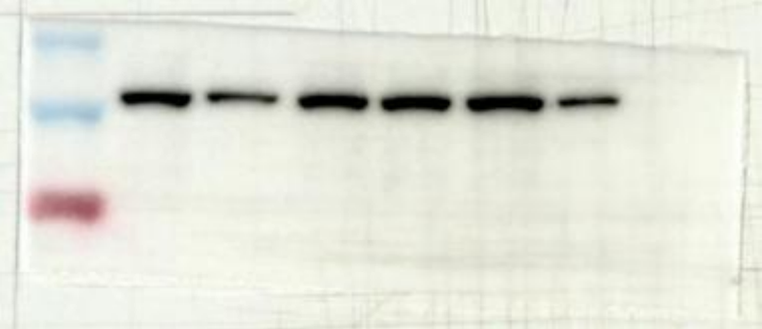

β-actin

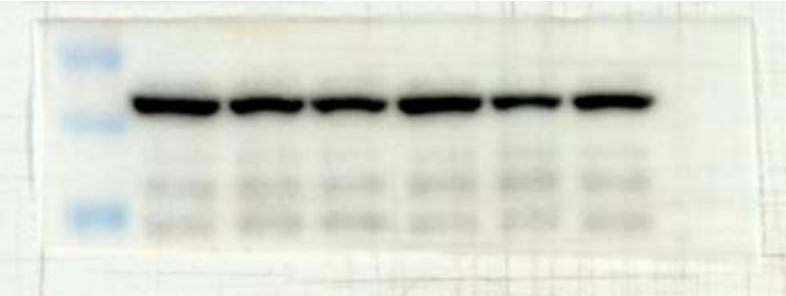

Figure 7D

Human 1-4 Cop1

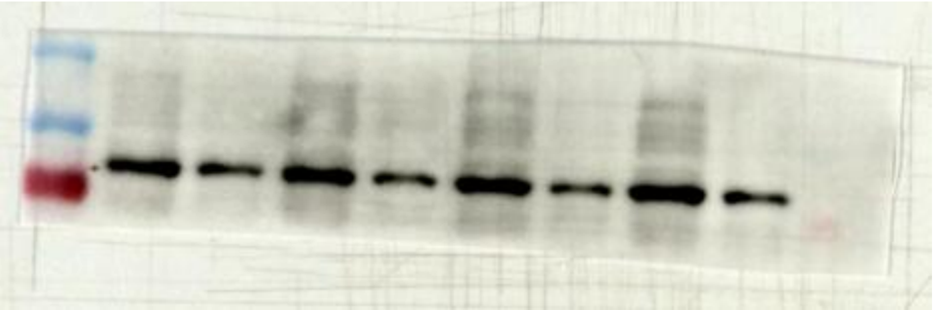

Human 1-4 β-actin

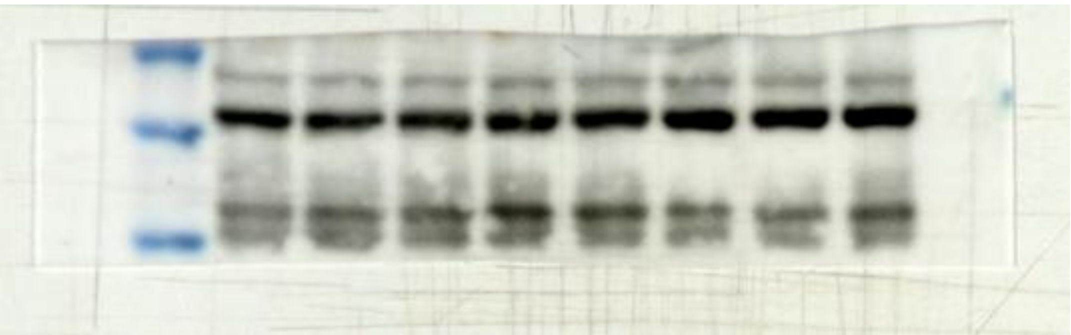

Human 5-8 Cop1

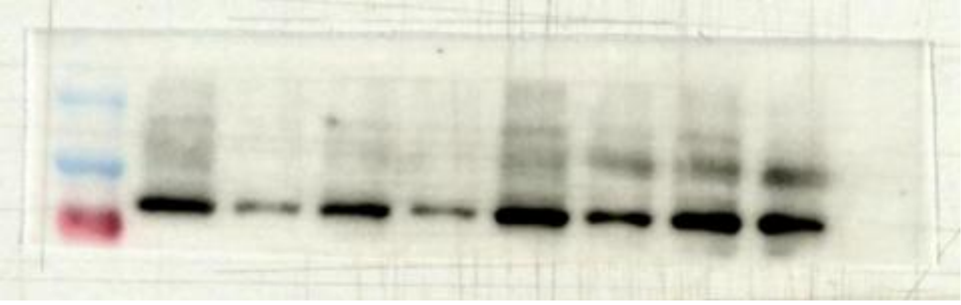

Human 5-8 β-actin

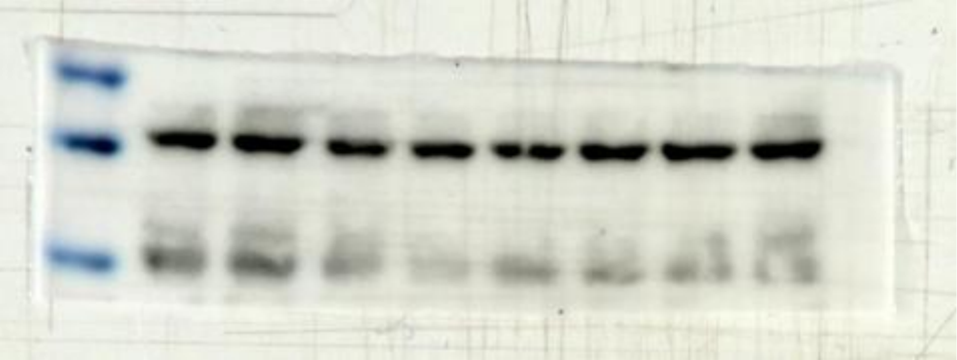

Human 9-12 Cop1

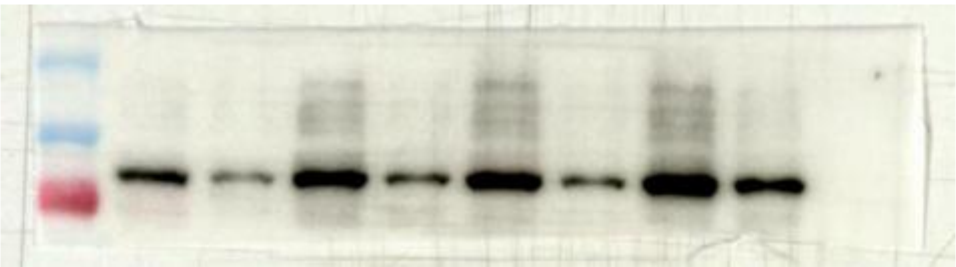

Human 9-13 β-actin

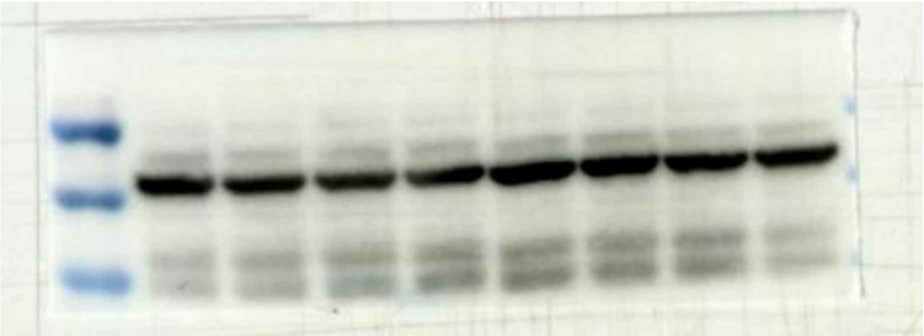

Human 13-16 Cop1

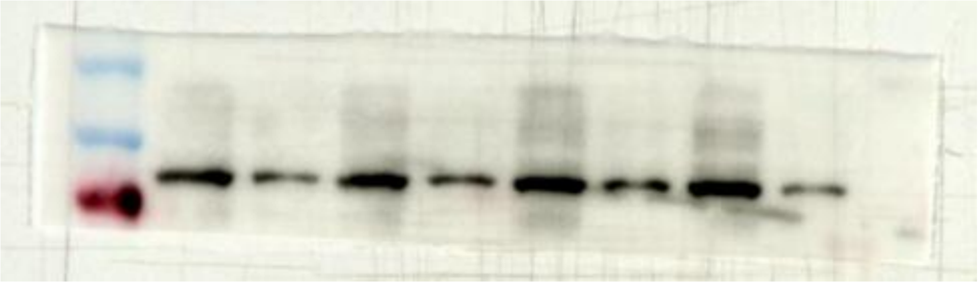

Human 13-16 β-actin

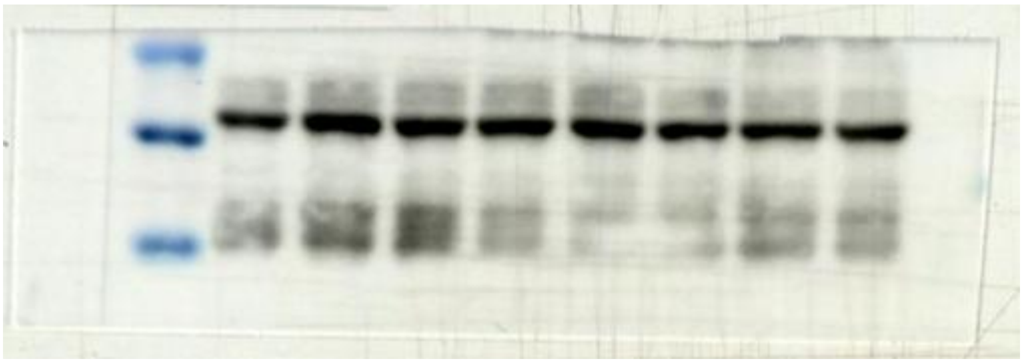

Figure 7E

Cop1

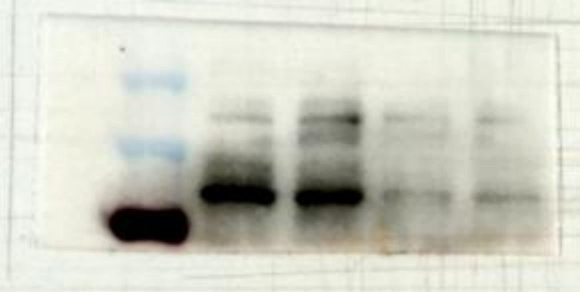

β-actin

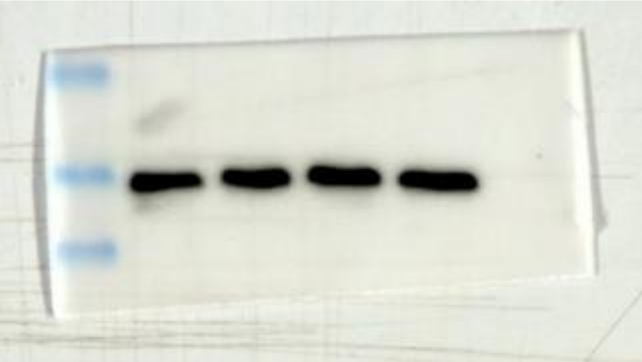

Figure 7F

Flag

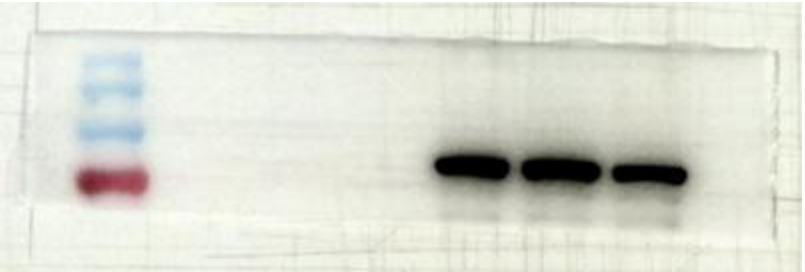

β-actin

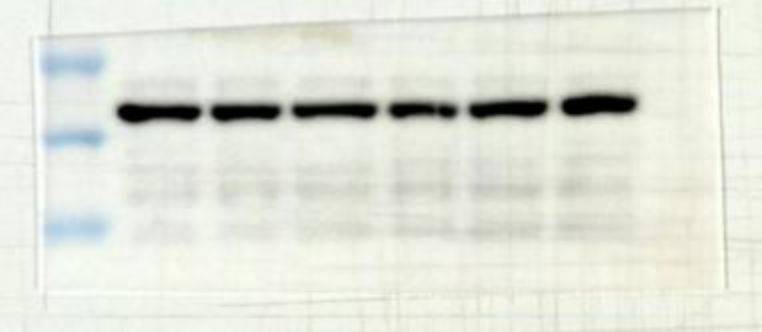

Figure 7G

Bax

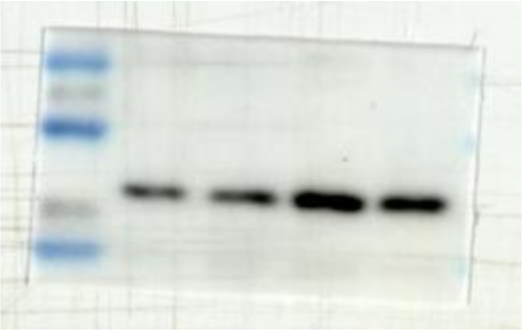

Bcl2

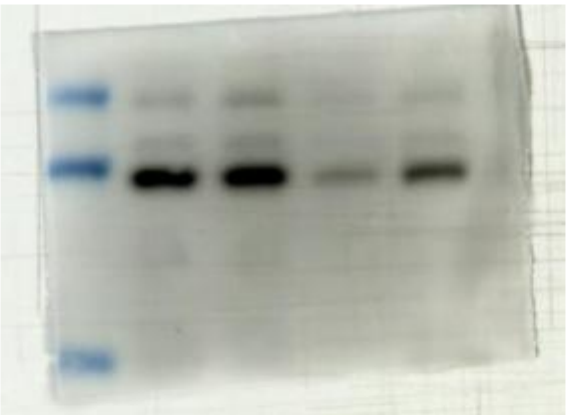

C-caspase3

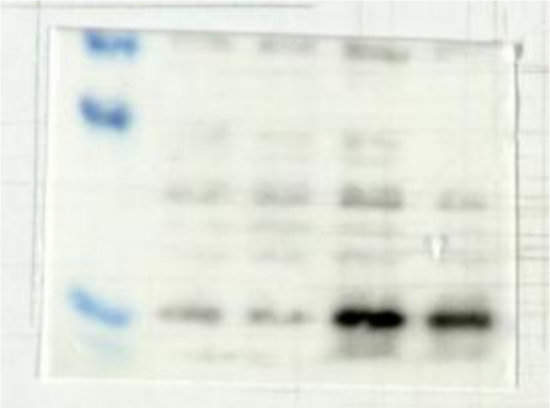

β-actin

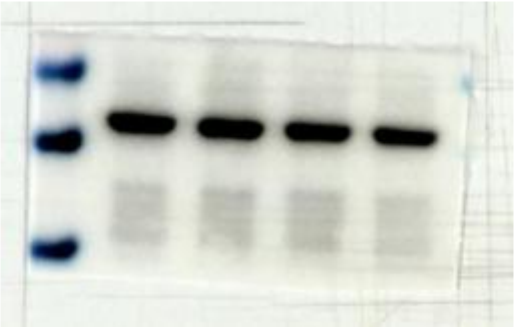

Figure 7I

IKKβ

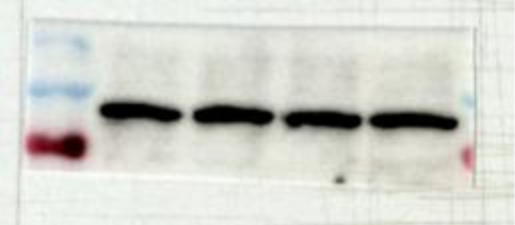

p-IKKβ

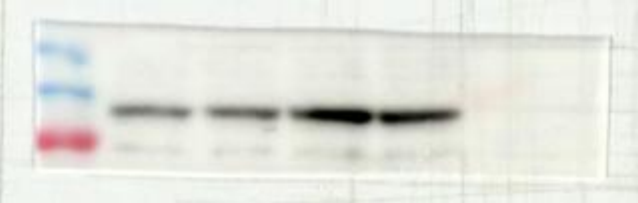

IKBα

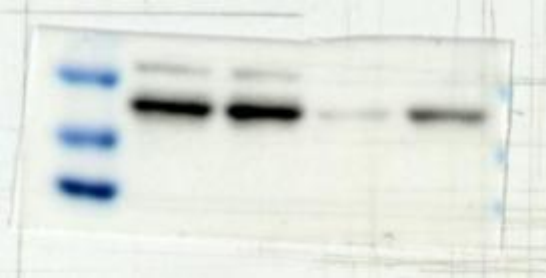

p65

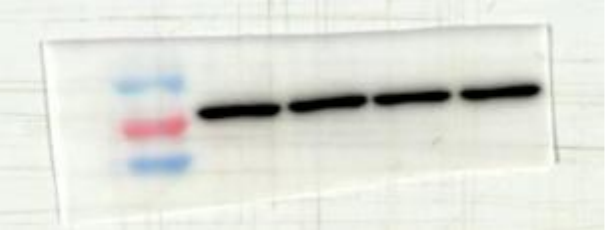

p-p65

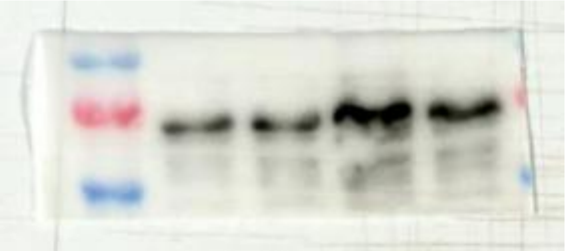

β-actin

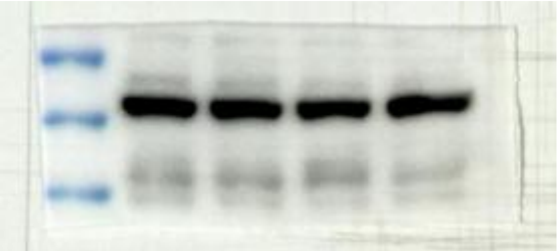

Figure 8

Figure 8A

IP:HA HA

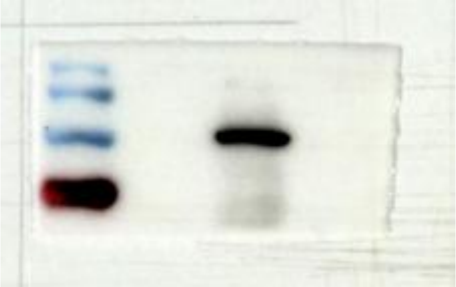

IP:HA Flag

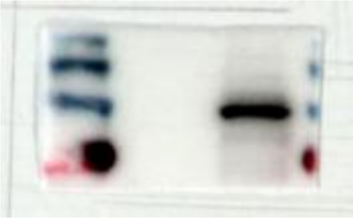

IP: HA input HA

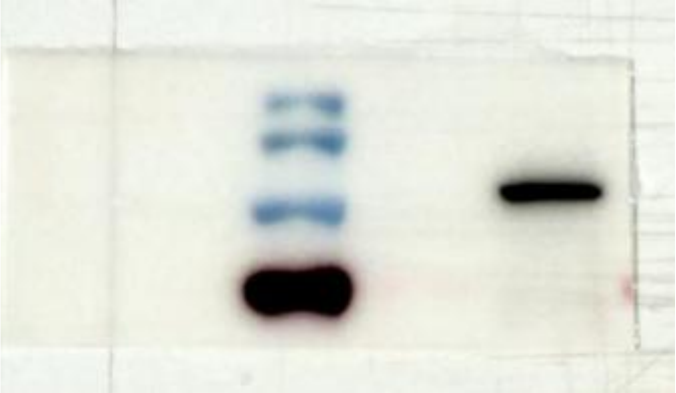

IP: HA input Flag

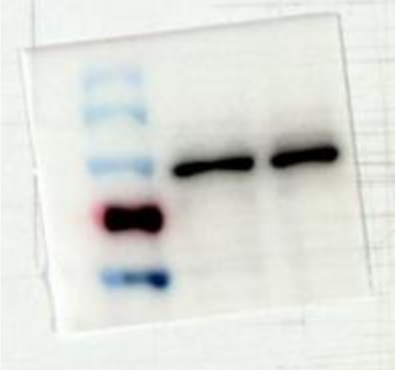

IP:Flag HA

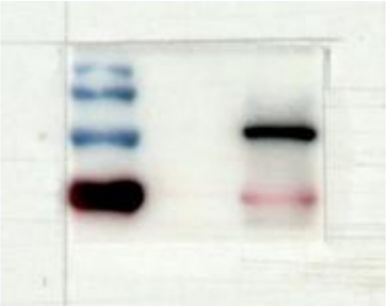

IP:Flag Flag

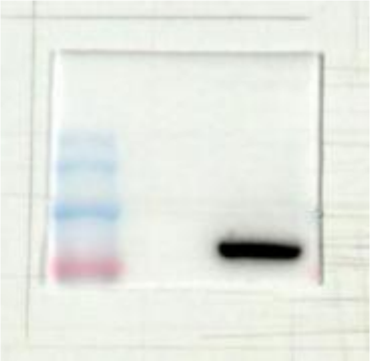

IP:Flag input HA

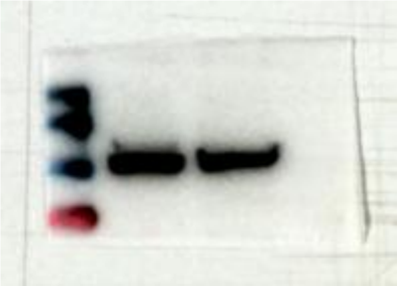

IP:Flag input Flag

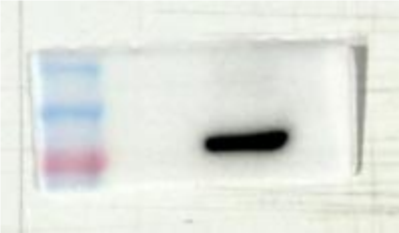

Figure 8B

IP:HA HA

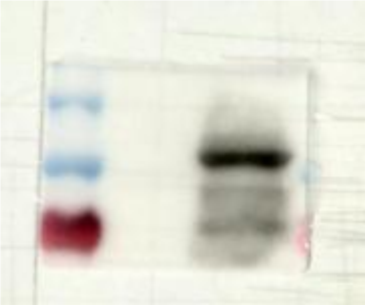

IP:HA Flag

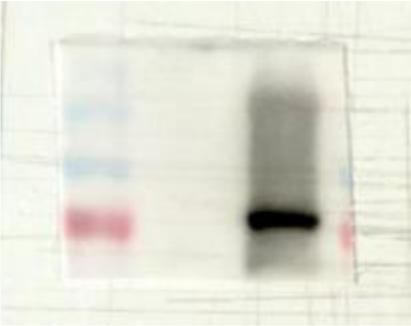

IP:HA input HA

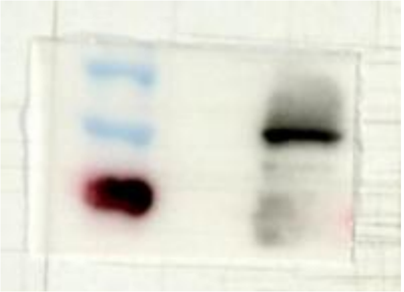

IP:HA input Flag

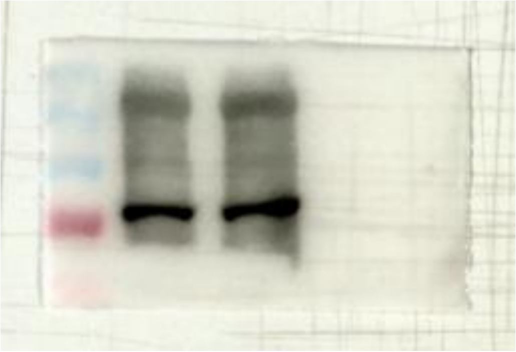

IP:Flag HA

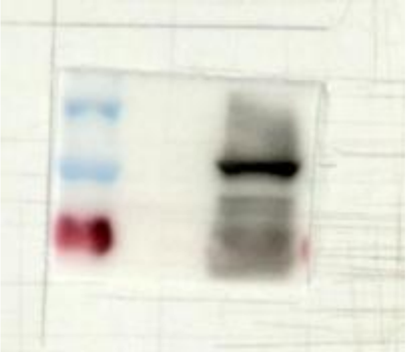

IP:Flag Flag

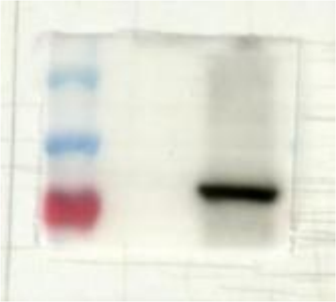

IP:Flag input HA

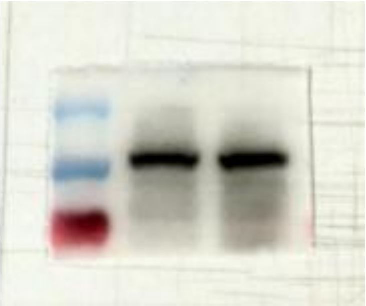

IP:Flag input Flag

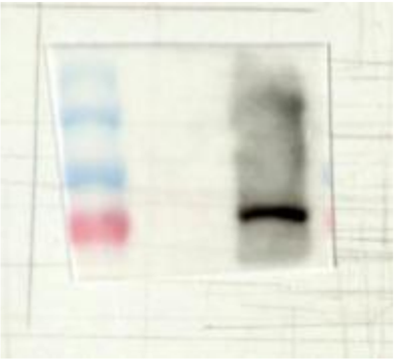

Figure 8D

Rhbdf2

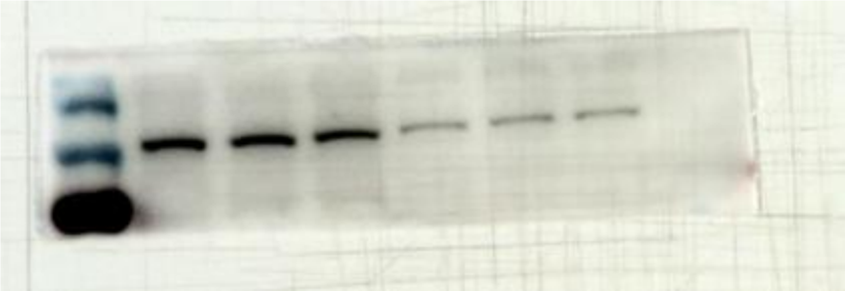

TAK1

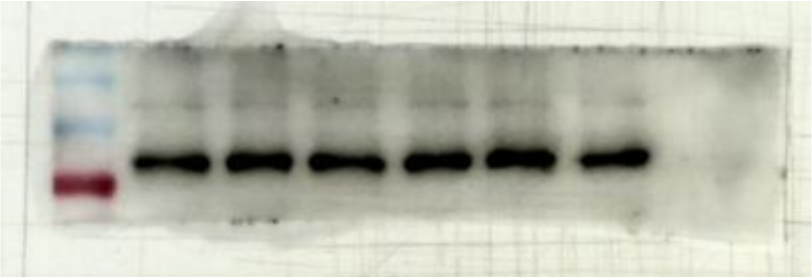

p-TAK1

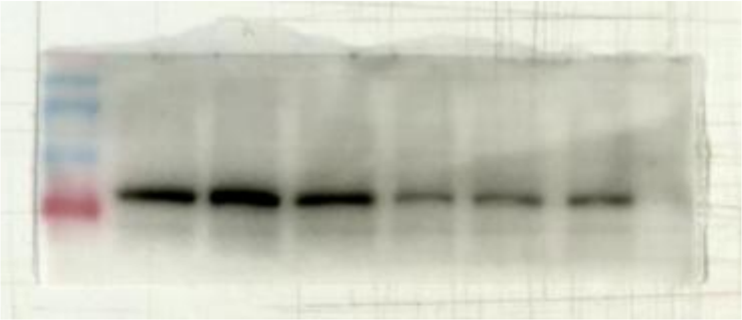

Flag

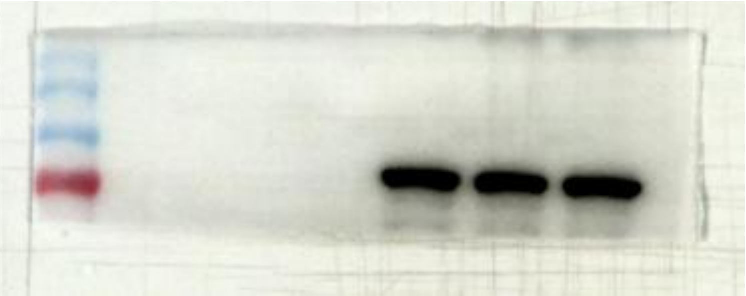

β-actin

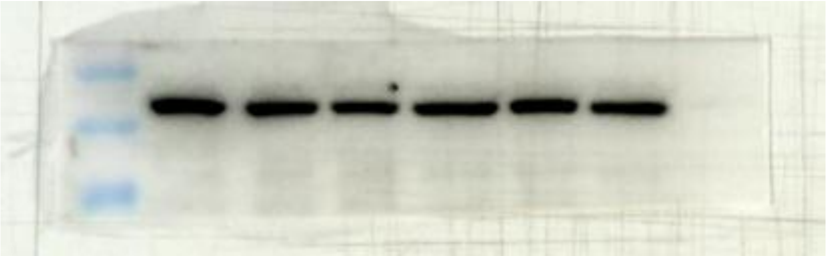

Figure 8E

Rhbdf2

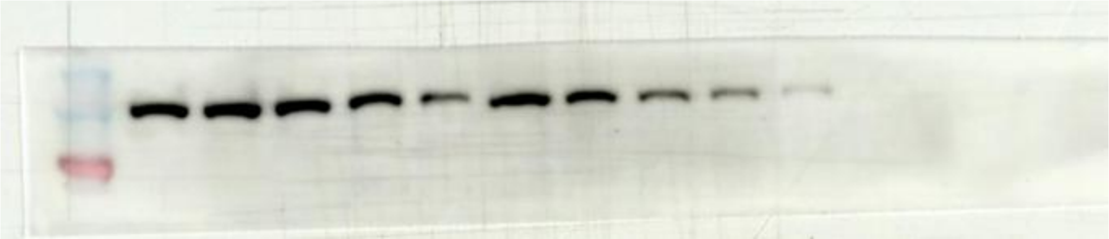

Flag

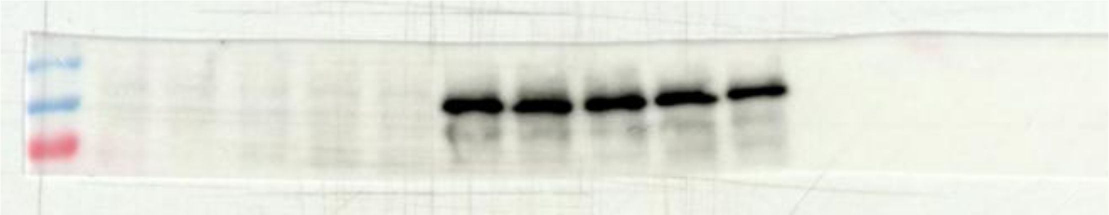

β-actin

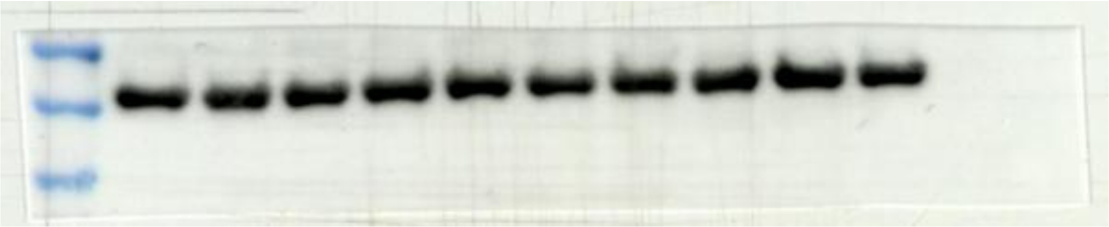

Figure 8F

Rhbdf2

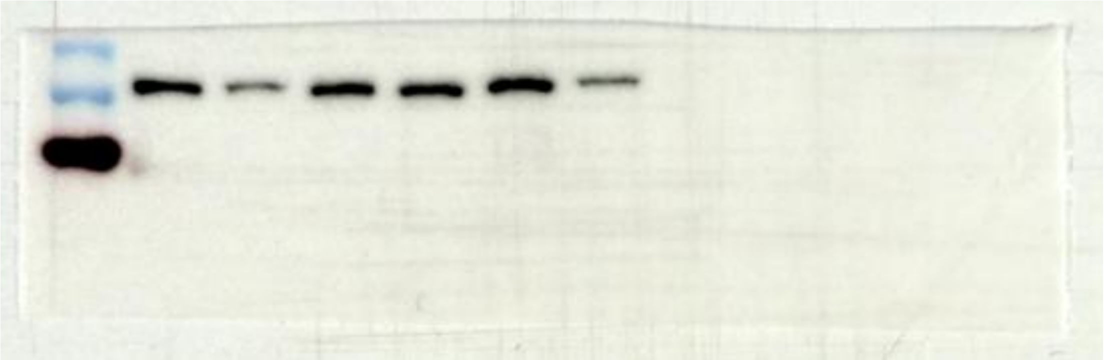

Flag

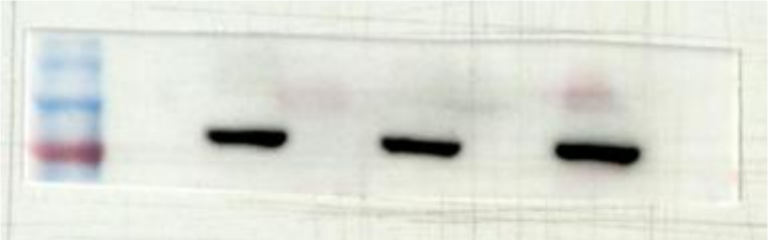

β-actin

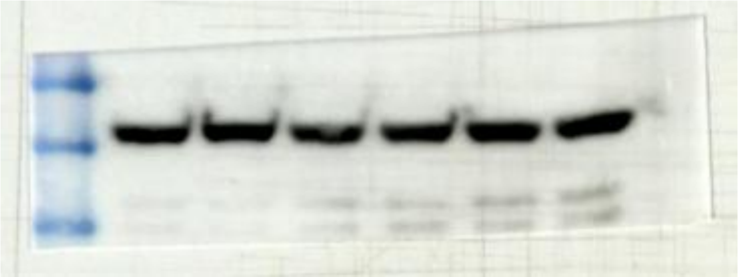

Figure 8G

IP:HA Myc

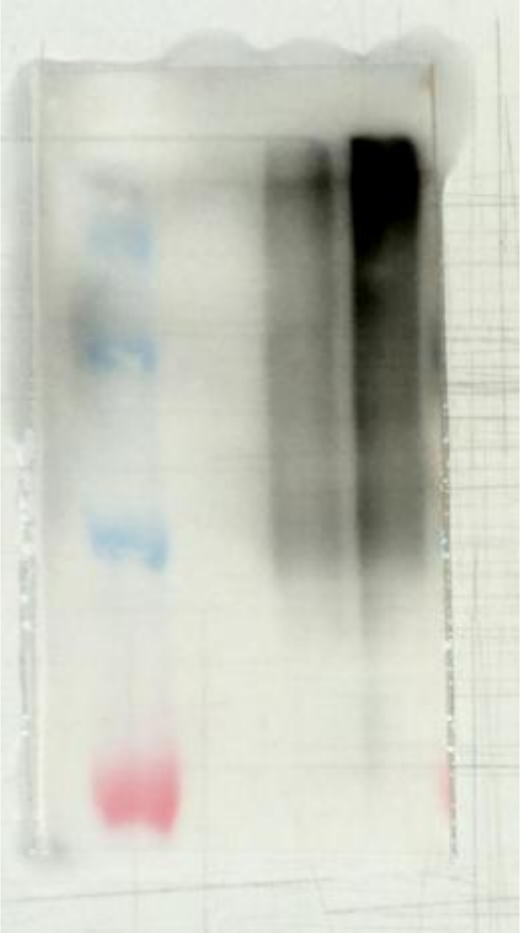

IP:HA HA

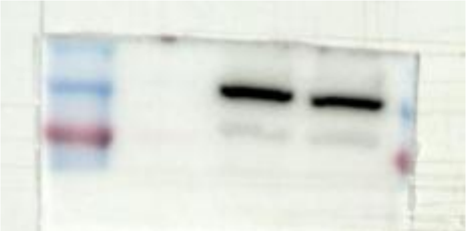

IP:HA input Flag

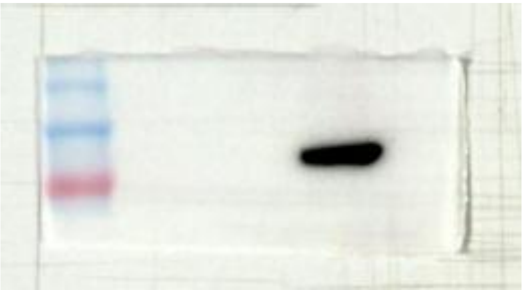

IP:HA input HA

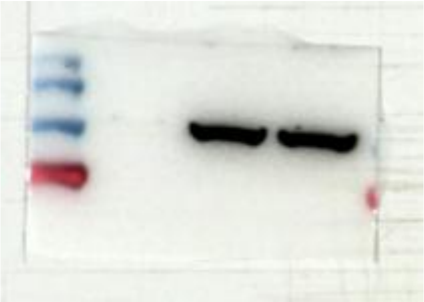

Figure 8H

IP:HA Myc

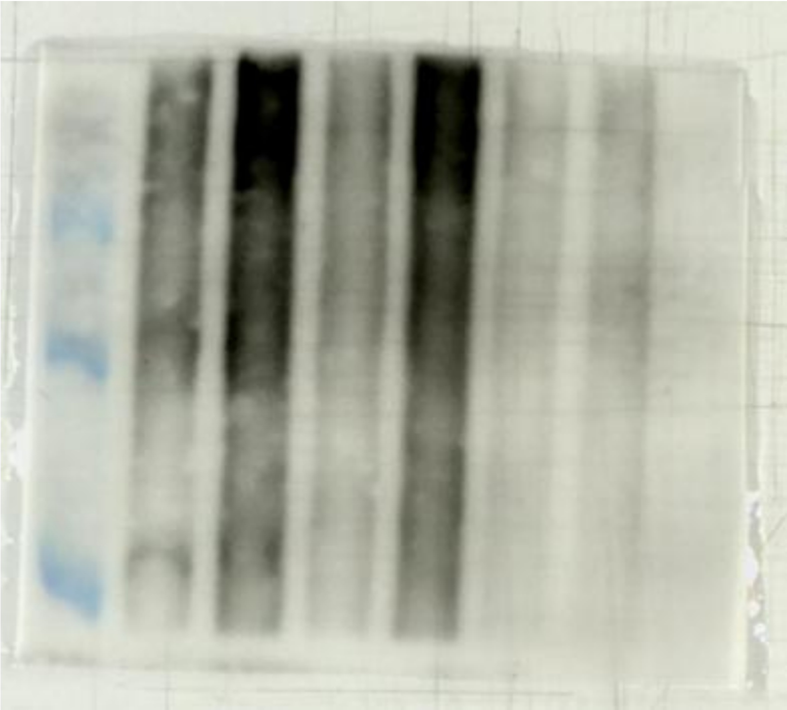

IP:HA HA

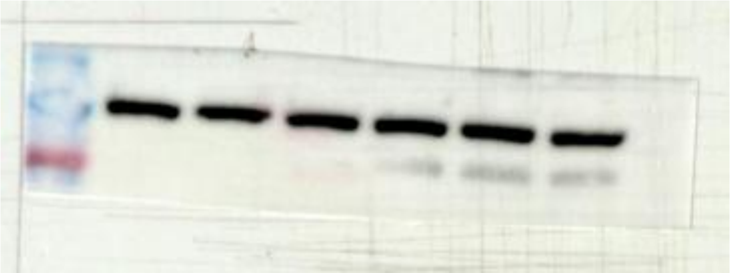

IP:HA input Flag

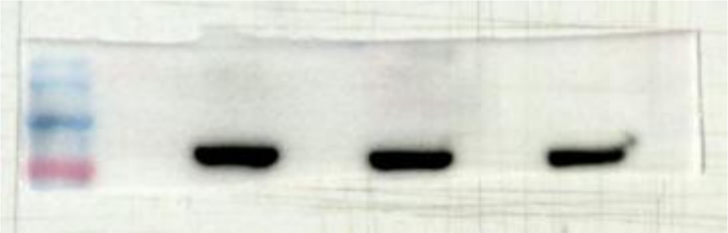

IP:HA input HA

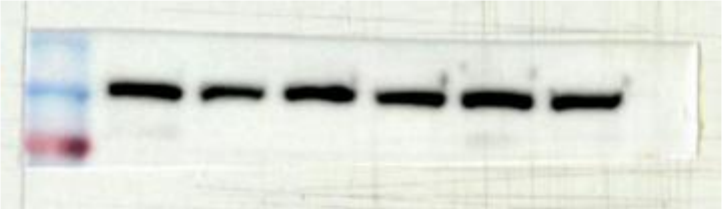

Figure 9

Figure 9B

Bax

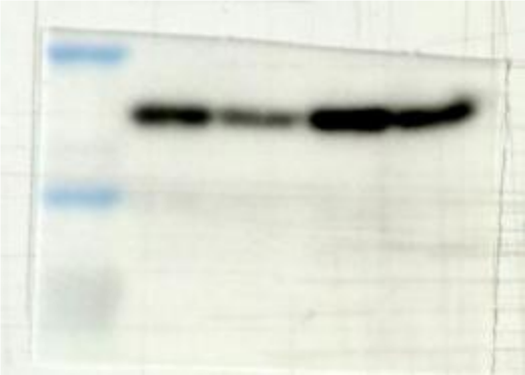

Bcl2

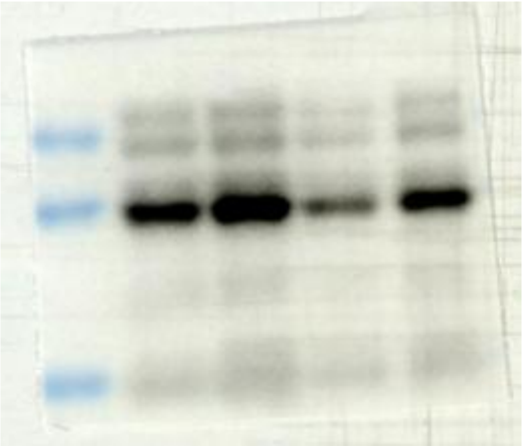

C-caspase3

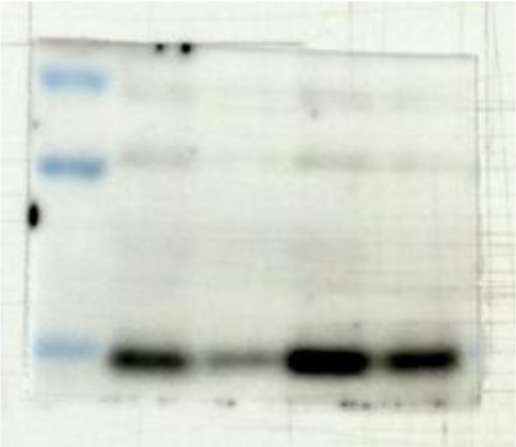

β-actin

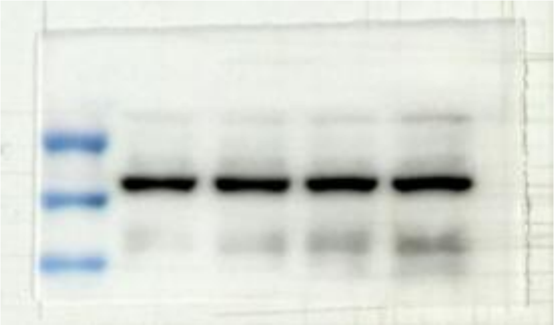

Figure 9D

IKKβ

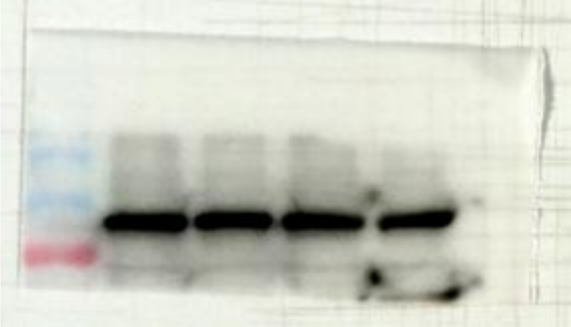

p-IKKβ

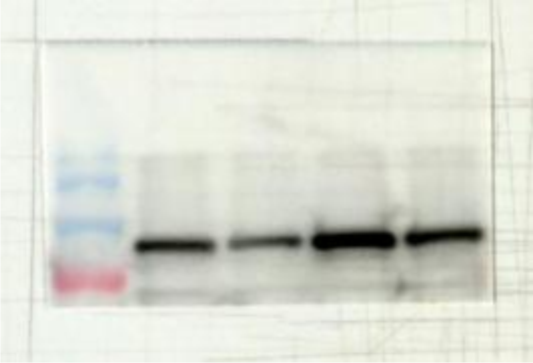

IKBα

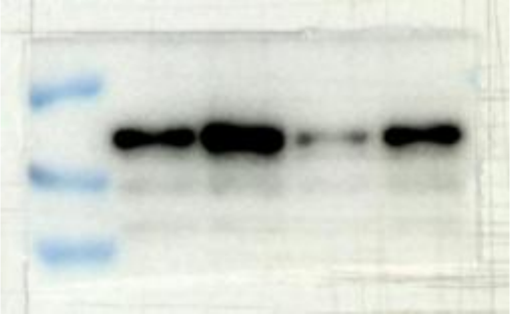

p65

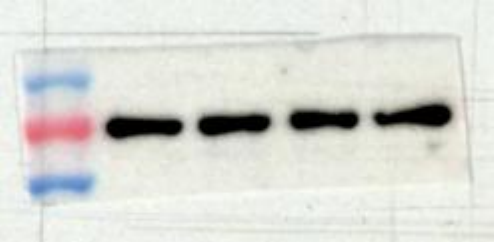

p-p65

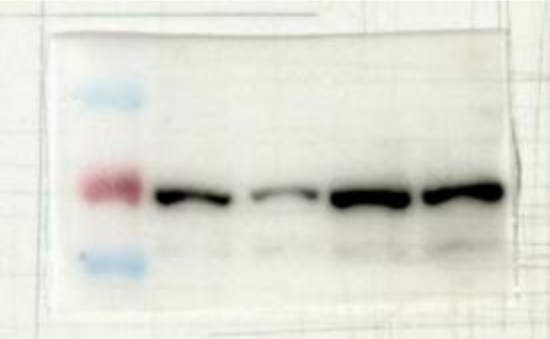

β-actin

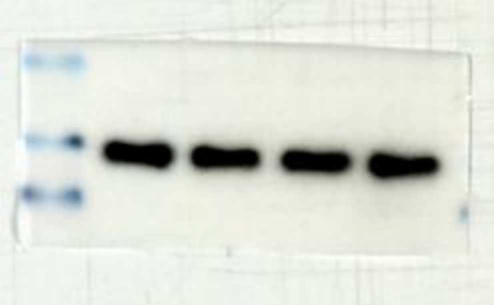

Figure 9E

TAK1

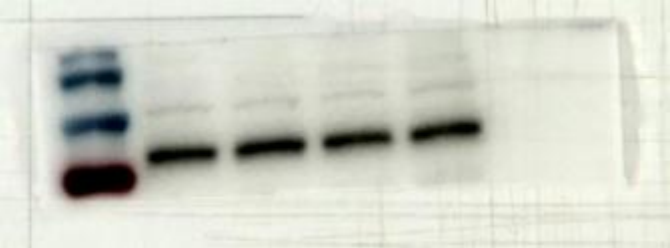

p-TAK1

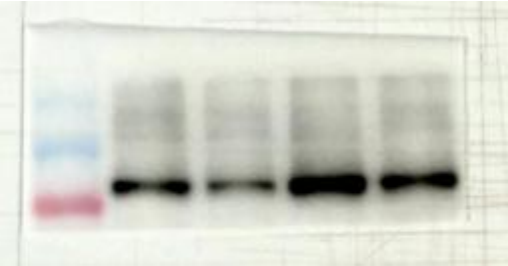

JNK

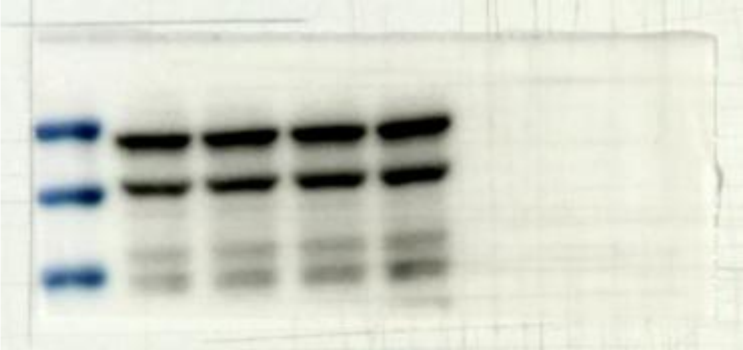

p-JNK

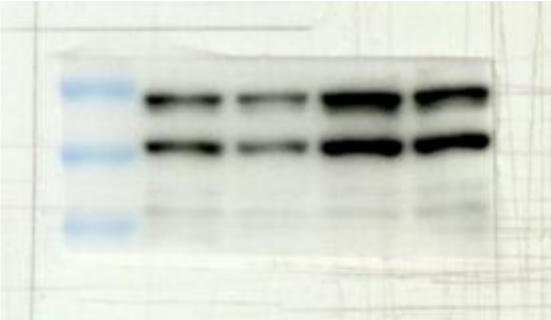

p38

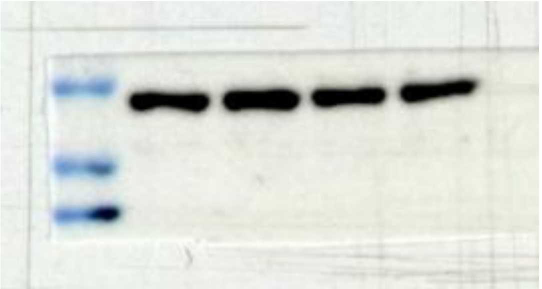

p-p38

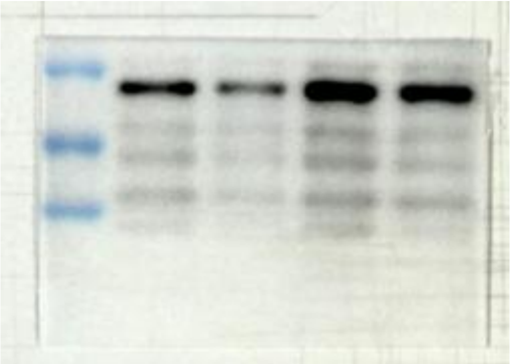

β-actin

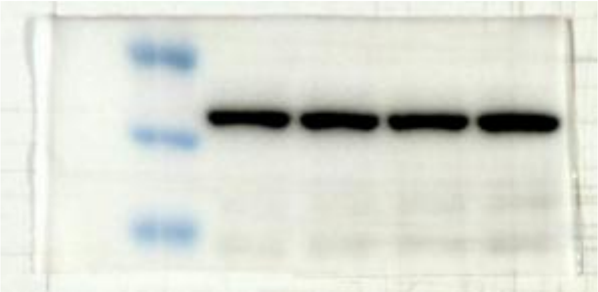

Supplement: Supplementary Material [file mmc1.pdf]
